# Causal associations between environmental factors and risk of IgA nephropathy and membranous nephropathy: a bidirectional Mendelian randomization and mediation analysis

**DOI:** 10.1080/0886022X.2025.2486620

**Published:** 2025-04-09

**Authors:** Chunmin Li, Qian Wen, Yanxia Zhang, Jun Wu

**Affiliations:** Department of Nephrology, Tongren Hospital of Wuhan University (Wuhan Third Hospital), Wuhan University, Wuhan, P.R. China

**Keywords:** Environmental exposures, IgA nephropathy, membranous nephropathy, mendelian randomization, mediation analysis, risk factors

## Abstract

**Aims:**

IgA nephropathy (IgAN) and membranous nephropathy (MN) have intricate etiologies that are poorly understood. This study aimed to investigate the impact of genetically predicted environmental factors on IgAN and MN.

**Methods:**

We used bidirectional two-step Mendelian randomization (MR) analysis utilizing large-scale genome-wide association study (GWAS) data to investigate the relationships between 68 environmental exposures and IgAN and MN. The main method is inverse variance weighted (IVW). Sensitivity analyses were conducted to validate the causal estimates. Furthermore, the two-step MR was used to explore possible mediating effects.

**Results:**

A total of 20 significant causal associations were identified. Genetically predicted educational attainment (EA), average household income, gluten-free diet, cheese intake, fresh fruit intake, cognitive performance, and intelligence were associated with a reduced risk of IgAN (*p* < 0.05); whereas frequency of alcohol consumption, insomnia, triglycerides, transferrin saturation, percentage body fat, body mass index (BMI), waist circumference, and blood pressure were associated with the risk of IgAN (*p* < 0.05). Genetically predicted EA and moderate to vigorous physical activity were associated with a reduced risk of MN (*p* < 0.05); on the other hand, beef intake, waist-to-hip ratio, and nitrogen oxides were associated with the risk of MN (*p* < 0.05). In addition, we observed that insomnia, BMI, and waist circumference partially mediated the causal link between EA and IgAN, with mediation proportions of 12.52%, 11.06%, and 9.93%, respectively.

**Conclusions:**

This study provides novel evidence of causal relationships between 20 genetically predicted environmental factors and the risk of IgAN and MN. These findings may inform targeted prevention strategies and contribute to improved disease risk assessment.

## Introduction

IgA nephropathy (IgAN) and membranous nephropathy (MN) are the predominant types of glomerulonephritis [1], increasing patients’ economic burden and drastically lowering quality of life. IgAN has an incidence of 25 per million population yearly [2] and is the primary contributor of kidney failure in patients under 40, with 30–40% of adult patients progressing kidney failure over a 20–30 year span [3]. Membranous nephropathy (MN) is the most common cause of nephrotic syndrome in adults, with an incidence of 10–12 per million in North America and 2–17 per million in Europe [4]. Nearly one-third of MN patients eventually progress to end-stage renal disease (ESRD), and within 10 years post-diagnosis, at least half of these patients succumb to the disease or necessitate renal replacement therapy [5]. Consequently, elucidating the etiology and identifying risk factors for IgAN and MN may offer vital insights for the development of preventative and therapeutic strategies.

The pathogenesis of IgAN and MN, as autoimmune diseases, remains incompletely understood. The prevalent ‘multiple-hit’ theory falls short in explaining IgAN’s evident geographic distribution, racial diversity, and clinical heterogeneity [3]. In addition to the effects of genetics and epigenetics, non-genetic factors (environmental factors) should be considered. Both external (e.g., pollutants, diet, lifestyle) and internal (e.g., metabolic factors, gut microbiome, hormones, inflammation) environmental exposures have been implicated in the incidence and progression of IgAN [6,7]. Roughly 80% of MN patients present with no identifiable underlying etiology (primary MN), with the remaining cases attributed to medications or other conditions [4]. Similarly, it has been shown that the interaction between genetic susceptibility and environmental factors plays an important role in the pathogenesis of MN, encompassing exposures such as PM2.5 [7], occupational hydrocarbons, and *Helicobacter pylori* infection [8]. Early intervention that targets these environmental factors may be able to prevent and postpone the onset and progression of disease. Nonetheless, there exists a divergence of opinions regarding the involvement of specific environmental exposures in these two glomerular diseases. The majority of existing evidence is based on observational studies, which inherently cannot fully exclude biases resulting from confounding and reverse causation. The various exposure factors are intricately intertwined, adding to the complexity of causal associations. A higher level of evidence is required to clarify the effects of environmental exposures on IgAN and MN.

Mendelian randomization (MR), utilizing genetic variation as an instrumental variable, is an effective new strategy for establishing causal relationships between exposure and outcome. Compared to observational studies, there are several advantages. It overcomes the limitation of requiring large samples and long-term follow-up to detect endpoints and can be used to investigate exposures that have expected adverse effects on disease. Additionally, during meiosis and fertilization, genetic variations are randomly assigned, which avoids confounding factors and reverse causation to some extent [9]. The objective of this study was to use MR method to evaluate the causal association between five environmental exposures and the development of two glomerular diseases, IgAN and MN. Previous MR analyses have revealed that external environmental factors (e.g., educational attainment) can influence disease progression by changing an individual’s internal environmental factors (e.g., personal behavior and metabolism) [10,11]. We performed a two-step MR analysis to dissect the mediating role of individual behavior, metabolism in the association of the external environment with disease, offering novel insights into their prevention.

## Methods

### GWAS data sources for exposure factors

Environmental exposure factors are classified into five categories: socioeconomic, air pollution, personal behavior, metabolic factors, and physical examination. The results of Genome-wide association study (GWAS) research with larger sample sizes and more recent investigations were chosen as the source of instrumental variables for exposure. Instrumental variables (IVs) were obtained from the Integrative Epidemiology Unit (IEU) OpenGWAS database (IEU OpenGWAS project (mrcieu.ac.uk)) and the GWAS Catalog database (GWAS Catalog (ebi.ac.uk)). Due to the amount of GWAS data involved, we present the classification, GWAS ID, sample size, major consortium/cohort, author (year), and PubMed ID information for each exposed GWAS in the supplementary material.

**Table 2.** Effect of each mediator on IgAN with adjustment for educational attainment (EA).

Mediator	Method	nsnp	b	se	OR (95%CI)	*p*	Heterogeneity	Global Test
Behavioral factors								
Cheese intake	IVW	40	−0.175	0.124	0.839 (0.658–1.070)	0.158	0.785	0.774
Fresh fruit intake	IVW	29	−0.310	0.212	0.733 (0.484–1.111)	0.143	0.651	0.657
Alcohol intake frequency	IVW	60	0.063	0.061	1.065 (0.944–1.202)	0.304	0.891	0.891
insomnia	IVW	21	0.506	0.185	1.657 (1.152–2.383)	0.006	0.909	0.922
Physical measurements								
Body mass index (BMI)	IVW	364	0.127	0.041	1.13 5(1.047–1.230)	0.002	0.285	0.293
Body fat percentage	IVW	301	0.035	0.066	1.036 (0.911–1.178)	0.592	<0.001	0.001
Waist circumference	IVW	279	0.155	0.052	1.168 (1.056–1.293)	0.002	0.388	0.403
Hip circumference	IVW	341	0.127	0.043	1.135 (1.044–1.235)	0.003	0.002	0.003

### GWAS data sources for outcome

The GWAS summary data for IgAN and the primary MN traits were downloaded from the Integrated Epidemiology Unit (IEU) OpenGWAS database. The IgAN (GCST90018866) dataset is derived from a meta-analysis of 477,784 European samples (15,587 IgAN cases and 462,197 controls) and 24,182,646 single-nucleotide polymorphisms (SNPs) conducted by the UK Biobank and Finnish Genetics. The primary MN (ebi-a-GCST010005) were acquired from five European cohorts totaling 7,979 individuals (2,150 primary MN cases and 5,829 controls) and 5,327,688 single nucleotide polymorphisms (SNPs) conducted by the Kiryluk Lab [12]. All cases of IgAN and primary MN were diagnosed by renal biopsy, and any suspected cases secondary to autoimmune disease, drugs, infection, or malignancy were excluded. The supplementary material reports details of the two GWAS data.

The GWAS data for this study came from European populations. The original literature describes data sample collection, confounding control, and statistical methods, and ethical permission has been acquired. Exposure factor GWAS data were almost taken from the MRC Integrative Epidemiology Unit, IgAN data from the UK Biobank and FinnGen databases, and MN data from the Kiryluk Lab. We used data from different sources to minimize sample overlap to ensure robustness. The Strengthening the Reporting of Observational Studies in Epidemiology - Mendelian Randomization (STROBE-MR) checklist is available in Supplementary material.

### Study design

**Figure 1.**
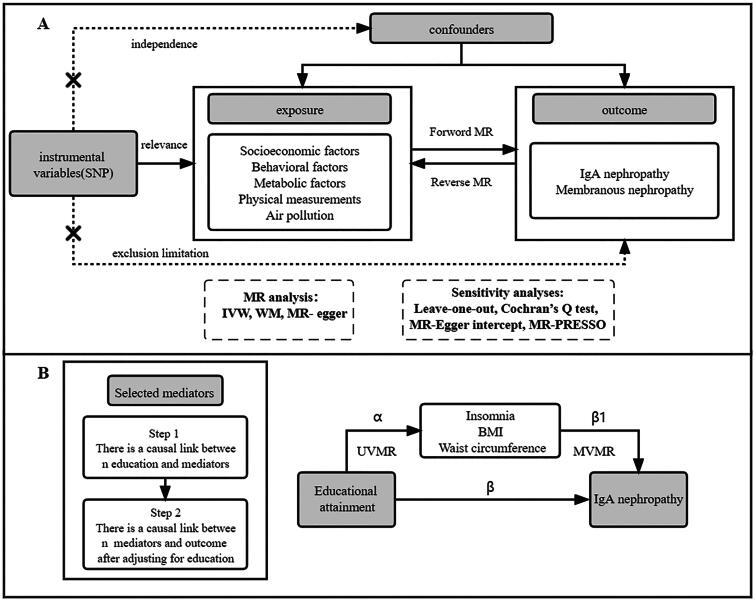
Overview of the study design. A depicts this mendelian randomization (MR) investigation of the causal relationship between environmental factors and two glomerular diseases. B depicts the mediated MR analysis.

In this two-sample MR study, we performed univariable, multivariable, bidirectional, and two-step MR analyses to investigate the association between environmental factors and IgAN and MN. A total of 68 main risk factors were selected, which were divided into 5 categories: socioeconomic factors, air pollution, behavioral factors, metabolic factors, physical measurements. MR analyses were based on three basic assumptions: the instrumental variable SNPs were strongly linked with exposure factors, but not with other confounders, and they only influenced outcomes *via* the exposure pathway (See Figure 1).

### Selection of instrumental variables

Instrumental variable screening criteria: SNP with *p*-value < 5 × 10^−8^ and minor allele frequency (MAF) > 0.01 were extracted. The linkage disequilibrium (LD) SNPs were removed. The linkage disequilibrium (r^2^) was 0.001 and the genetic distance was 10,000 kb. SNPs were removed if they were associated (*p*-value < 0.001) with the outcomes. Excluding weak IVs with *F* < 10, F = R^2^ (n – k − 1)/k (1 – R^2^). Finally, the GWAS datasets for exposure and outcome were harmonized to remove palindromic SNPs with intermediate allele frequencies (>0.42) or SNPs with incompatible alleles.

### Selection of mediator variables

Based on the literature review, we selected mediators that may play a role in the pathway from education attainment (EA) to outcome, this included metabolic factors (BMI, waist circumference, hip circumference, percent body fat), lifestyle habits (frequency of alcohol consumption, diet habits), and stressful events (insomnia). Selection criteria for mediator variables: there is a causal relationship between exposure and mediator. Mediator were linked to outcome, and this correlation persisted even adjustment of EA (See Figure 1).

### Statistical analysis

The causative connection between five types of environmental factors and membranous and IGA nephropathy was estimated using three different methods: weighted median (WM), MR-egger, and inverse variance weighted (IVW). The overall estimates using the IVW were used as main effects, with WM and MR- egger as complements. Additional sensitivity studies to evaluate the causal link between each group of exposures and outcome. Outliers were detected using leave-one-out analysis, and heterogeneity was assessed using the Cochran’s Q test. Where heterogeneity existed, it was evaluated using the IVW random effects model. The MR-Egger intercept method was used to test for horizontal pleiotropy. When *p*-value < 0.05, it indicates horizontal pleiotropy. The MR-Pleiotropy Residual Sum and Outlier (MR-PRESSO) was used to remove outlier SNPs and assesse whether excluding outlier SNPs affects causal estimations. The bi-directional MR analysis was used to determine if there was reverse causality in the observed causal estimates. Two-step Mendelian randomization was used to assess mediation effects. The first step was to use UVMR to estimate the causal effect of genetically determined exposure on mediator (α). The second step used MVMR adjusting for exposure factors to estimate the causal effect of mediator (β1) on outcome. The mediation effect is calculated with the product of coefficients method (α × β1). Proportion of mediating effect is calculated as the outcome of the mediating effect (α × β1) was divided by the total effect (β) estimated by the UVMR. The standard deviation of the mediating effect was calculated using the Delta method and confidence intervals were further calculated. The bootstrap method with 1000 iterations was used to calculate 95% confidence intervals for the proportions of mediated effects. All MR analyses were performed using the R package ‘TwoSampleMR’, ‘MRPRESSO’, ‘MVMR’ in the R version 4.3.2. The forest plot was produced using the ‘forestploter’ software package. Scatterplots and funnel plots are generated from the ‘TwoSampleMR’ software package.

## Results

### Baseline characteristics

All IVs in the study had F values greater than 10, ranging from 27 to 808, and the number of SNPs ranged from 2 to 300, see the supplementary file for details. Our study investigated the relationship between 68 environmental exposures and two glomerular diseases. We identified a total of 20 causal relationship pairs. Eight novel relationships were identified, including causal links between IgAN and cheese intake, fresh fruit consumption, insomnia, cognitive performance, intelligence, and transferrin saturation. Additionally, we observed a causal association between MN and beef intake, as well as moderate to vigorous physical activity. We validated 12 causal relationships from prior observational studies and MR analyses, encompassing IgAN’s associations with EA, household income, alcohol consumption frequency, gluten-free diet, triglyceride levels, BMI, waist circumference, body fat percentage, and blood pressure. Associations of MN with EA, waist-to-hip ratios, and NO were also included. In addition, we found that insomnia, BMI, and waist circumference partially mediated the causal relationship between education level and the risk of IgAN. For the positive findings results of the MR analyses, sensitivity analyses, and visualization plots are available in the Supplementary Information.

### Causal effects of environmental factors on IgAN

**Figure 2.**
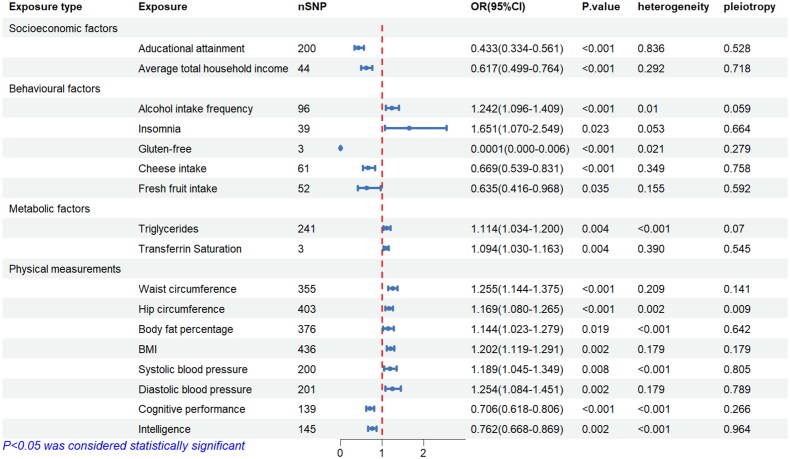
The causal effect of environmental factors on IgAN.

In terms of socioeconomic factors, univariate IVM analysis showed a causal relationship between genetically predicted increased educational attainment (EA) and decreased risk of IgAN (OR = 0.433, 95%CI: 0.334–0.560, *p* = 2.316 × 10^−10^). No evidence of pleiotropy (MR-Egger regression intercept: *p* = 0.836) or heterogeneity (Cochran’s Q test: *p* = 0.528) was detected. The genetically predicted higher average household economic income was causally linked to a reduced risk of IgAN (OR= 0.617, 95%CI: 0.499–0.764, *p* = 9.56 × 10^−6^). After removing two outliers, no pleiotropy (MR-Egger regression intercept: *p* = 0.292) or heterogeneity (Cochran’s Q test: *p* = 0.718) was detected (Figure 2). All of the above conclusions were supported by the WM method.

In terms of behavioral factors, the IVW method showed that genetically predicted higher frequency of alcohol intake and insomnia were causally associated with a higher risk of IgAN (OR= 1.242, 95%CI = 1.096–1.409, *p* < 0.001; OR= 1.651, 95%CI = 1.070–2.549, *p* = 0.023). Heterogeneity was observed with a Cochran Q-derived P value < 0.05 for frequency of alcohol intake, and no pleiotropy was detected (MR-Egger regression intercept: *p* = 0.059). Heterogeneity is allowed based on random-effects IVW. No heterogeneity (Cochran’s Q test: *p* = 0.053) or pleiotropy (MR-Egger regression intercept: *p* = 0.664) were detected for insomnia. Among dietary habits, genetically predicted gluten-free diet, cheese intake, and fresh fruit intake were found to be causally associated with a lower risk of IgAN (OR = 0.0001, 95%CI = 1 × 10^−6^–0.005, *p* < 0.001; OR = 0.669, 95%CI = 0.539–0.831, *p* < 0.001; OR = 0.635, 95%CI = 0.416–0.968, *p* = 0.035). Heterogeneity was observed for gluten-free diet (Cochran’s Q test: *p* = 0.021), but acceptable. No pleiotropy were detected for the three diets above (all MR-Egger regression intercept: *p* > 0.05) (Figure 2).

In terms of metabolic factors, MR analysis demonstrated genetically predicted higher triglyceride levels (OR= 1.114, 95%CI = 1.034–1.200, *p* = 0.004) and higher transferrin saturation (OR= 1.094, 95%CI = 1.030–1.163, *p* = 0.004) were associated with a higher risk of IgAN. No pleiotropy (MR-Egger regression intercept: *p* = 0.070) but heterogeneity (Cochran’s Q test: *p* < 0.05) was detected for triglycerides. No pleiotropy (MR-Egger regression intercept: *p* = 0.545) or heterogeneity (Cochran’s Q test: *p* = 0.389) were detected for transferrin saturation (Figure 2).

Regarding obesity traits, genetically predicted higher waist circumference (OR = 1.255, 95% CI = 1.144–1.375, *p* = 1.19 × 10^−6^), hip circumference (OR = 1.169, 95% CI = 1.080–1.265, *p* = 7.31 × 10^−5^), percentage of body fat (OR= 1.144, 95% CI = 1.023–1.279, *p* = 0.019), and BMI (OR = 1.202, 95% CI =1.119–1.291, *p* = 4.56 × 10^−7^) were causally associated with a higher risk of IgAN. Pleiotropy tests were non-significant except for hip circumference (MR-Egger intercept *p* = 0.009; global test *p* < 2^−4^). Nevertheless, the corrected MR-PRESSO result was still significant after removing outliers for hip circumference (outlier-corrected *p* = 0.0004). In the blood pressure category, MR analysis demostrated that higher systolic blood pressure (OR = 1.188, 95% CI = 1.045–1.349, *p* = 0.008) and diastolic blood pressure (OR = 1.254, 95% CI = 1.084–1.451, *p* = 0.002) were significantly associated with increased risk of IgAN. No pleiotropy was detected (MR-Egger regression intercept: *p* = 0.805; MR-Egger regression intercept: *p* = 0.789) for blood pressure category.

Regarding the subgroup of cognition, MR analysis showed that genetically predicted better cognitive performance (OR= 0.706, 95% CI = 0.618–0.806, *p* = 2.57 × 10^−7^) and intelligence level (OR= 0.762, 95% CI = 0.668–0.869, *p* = 5.25 × 10^−5^) were associated with a lower risk of IgAN, with no pleiotropy was detected (MR-Egger regression intercept: *p* = 0.266; MR-Egger regression intercept: *p* = 0.964) (Figure 2).

### Causal effects of environmental factors on MN

**Figure 3.**
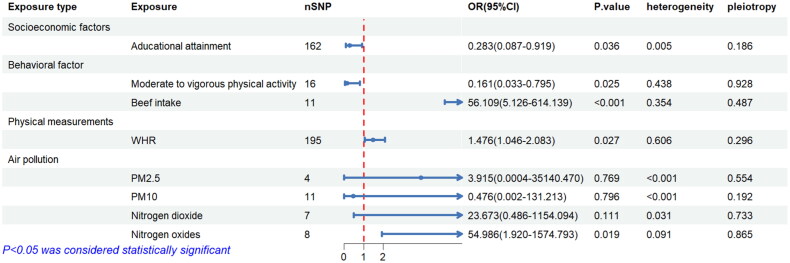
The causal effect of environmental factors on MN. WHR: waist-to-hip ratio.

Univariate MR analysis showed that in the socioeconomic factors, genetically predicted higher EA (OR = 0.283, 95%CI 0.087–0.919, *p* = 0.036) was suggestively associated with a lower risk of MN. Regarding the subgroup of behavioral factors, in MR analysis of moderate to vigorous physical activity to MN, we removed two outlier SNPs due to horizontal pleiotropy. The results showed that genetically predicted moderate to vigorous physical activity (OR = 0.161, 95%CI = 0.033–0.795, *p* = 0.025) was associated with low MN risk without heterogeneity (Cochran’s Q test: *p* = 0.478) and horizontal pleiotropy (MR-Egger regression intercept: *p* = 0.928). We also observed that beef intake positively associated with higher MN incidence (OR = 56.109, 95%CI = 5.126–614.139, *p* < 0.001), without evidence of heterogeneity or pleiotropy (Figure 3).

Regarding obesity traits, genetically predicted higher waist-to-hip ratios (adjusted for BMI) was causally associated with higher MN risk (OR = 1.476, 95%CI = 1.046–2.083, *p* = 0.027). No heterogeneity (Cochran’s Q test: *p* = 0.606) or pleiotropy (MR-Egger regression intercept: *p* = 0.296) was tested (Figure 3).

Among air pollutants, our UVMR analysis showed that NO was positively associated with a higher risk of MN incidence (OR = 54.986, 95% CI = 1.920–1.574 × 10^3^, *p* = 0.019), without evidence of heterogeneity (Cochran’s Q test: *p* = 0.091) or pleiotropy (MR-Egger regression intercept: *p* = 0.865). However, genetically predicted PM2.5 (*p* = 0.769), PM10 (*p* = 0.796) and nitrogen dioxide (*p* = 0.111) were not causally associated with MN risk, without evidence of pleiotropy (Figure 3).

### Reverse causality MR analysis

**Figure 4.**
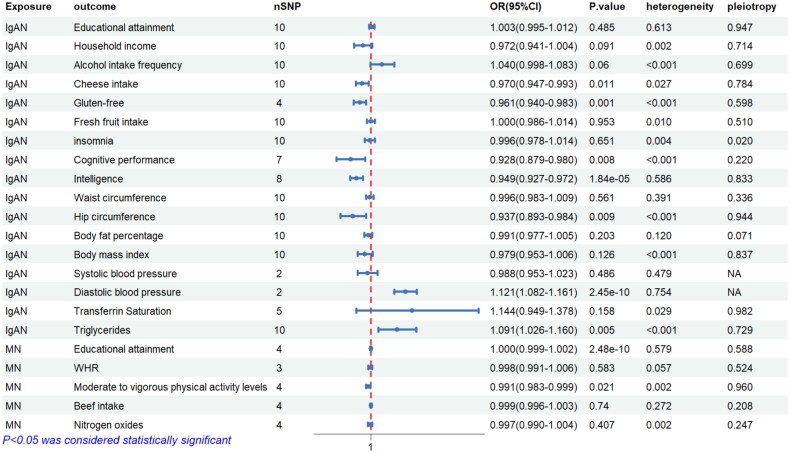
Inverse MR analysis of the effect of two glomerular diseases on environmental factors.

In the other direction, we further explored whether there was reverse causality in the significance of the UVMR results using IgAN or MN as the exposure factor. MR analysis showed a negative causal relationship between IgAN and gluten-free (OR = 0.970, 95%CI = 0.947–0.983, *p* < 0.001), cheese intake (OR = 0.970, 95%CI = 0.947–0.993, *p* = 0.011), cognitive performance (OR = 0.928, 95%CI = 0.879–0.980, *p* = 0.008), intelligence (OR = 0.949, 95%CI = 0.927–0.972, *p* < 0.001), hip circumference (OR = 0.937, 95%CI = 0.893–0.984, *p* = 0.009), a positive causal relationship between IgAN and triglycerides (OR = 1.091, 95%CI = 1.026–1.160, *p* = 0.005), without evidence of pleiotropy. No causal associations were found in MR analyses of IgAN with EA, household income, frequency of alcohol intake, fresh fruit intake, waist circumference, body fat percentage, body mass index (BMI), and transferrin saturation (*p* > 0.05), and there was no evidence of horizontal pleiotropy(*p* > 0.05). Genetically predicted IgAN was not causally associated with insomnia (*p* > 0.05), with pleiotropy detected (*p* = 0.004). The association of IgAN with blood pressure characteristics was not detected due to the small number of available SNPs. Using MN as the exposure, MR analysis showed that a negative causal association of MN on moderate to vigorous physical activity levels (OR = 0.991, 95% CI = 0.983–0.999, *p* = 0.021), without evidence of pleiotropy (*p* = 0.960). No causal associations were found in MR analyses of MN with EA, waist-to-hip ratio adjusted for BMI, beef intake, and nitrogen oxides (*p* > 0.05), and there was no evidence of horizontal pleiotropy (*p* > 0.05) (Figure 4).

### Intermediation MR analysis

**Figure 5.**
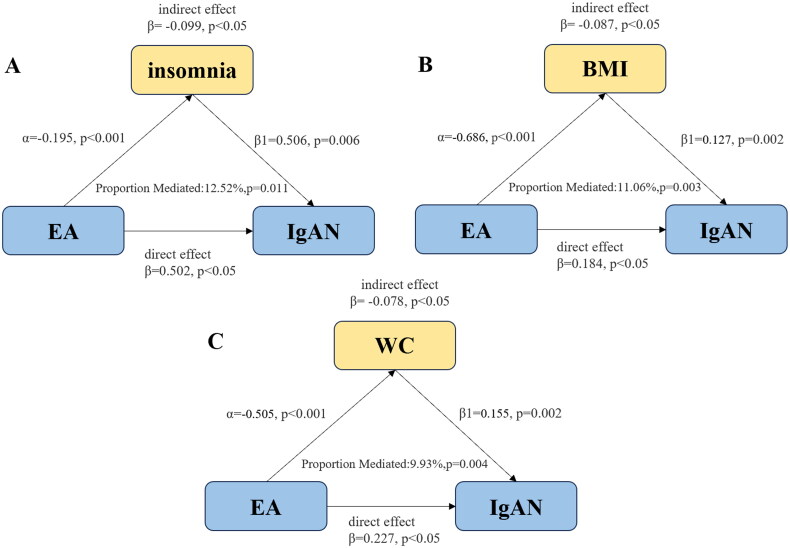
Path diagram of mediation analysis of the relationship between educational attainment (EA) and IgAN. A, B, and C represent the mediating roles of insomnia, body mass index (BMI), and waist circumference (WC), respectively.

Based on the literature, we investigated the mediators of the causative relationship between EA and IgAN. The first step of the UVMR analysis showed that EA was causally associated with frequency of alcohol intake (OR = 0.391, 95% CI = 0.343–0.445, *p* < 0.001), cheese intake (OR = 2.273, 95% CI = 2.088–2.475, *p* < 0.001), fresh fruit intake (OR = 1.198, 95% CI = 1.141–1.258, *p* < 0.001), percent body fat (OR = 0.591, 95% CI = 0.536–0.651, *p* < 0.001), BMI (OR = 0.504, 95% CI = 0.443–0.573, *p* < 0.001), and insomnia (OR = 0.823, 95% CI = 0.779–0.869, *p* < 0.001), without the evidence of pleiotropy(*p* > 0.05). (Table 1) The second step of MVMR analysis after adjusting for EA showed a positive causal relationship between insomnia (OR = 1.657, 95% CI = 1.152–2.383, *p* = 0.006), BMI (OR= 1.135, 95% CI = 1.047–1.230, *p* < 0.001), waist circumference (OR = 1.168, 95% CI = 1.056–1.293, *p* = 0.002), and there was no evidence of pleiotropy(*p* > 0.05). (Table 2) The mediating effect of insomnia, BMI, and waist circumference in the causal relationship between education and IgAN were −0.099 (β= −0.099, 95% CI= −0.175,−0.023, *p* = 0.011), −0.087 (β= −0.087, 95% CI= −0.145,−0.030, *p* = 0.003), −0.078 (β= −0.078, 95% CI= −0.132,−0.024, *p* = 0.004), respectively. Insomnia explained 12.52% (95% CI = 2.37%, 22.67%) of the total effect of EA on IgAN, while BMI explained 11.06% (95% CI = 3.44%, 18.67%), and waist circumference explained 9.93% (95% CI = 2.79%, 17.11%) of the total effect (Figure 5 and Table 3).

**Table 1.** UVMR Assessing the causal association between educational attainment (EA) and each mediator.

Mediator	Method	nsnp	b	se	OR (95%CI)	*p*	Heterogeneity	Egger intercept
Behavioral factors								
Gluten-free	IVW	135	0.001	0.011	1.000 (0.980–1.022)	0.926	0.572	0.546
Cheese intake	IVW	205	0.821	0.043	2.273 (2.088–2.475)	7.09 × 10^−80^	7.28 × 10^−44^	0.943
Fresh fruit intake	IVW	205	0.181	0.025	1.198 (1.141–1.258)	3.52 × 10^−13^	3.92 × 10^−53^	0.555
Alcohol intake frequency	IVW	205	−0.940	0.066	0.391 (0.343–0.445)	1.64 × 10^−45^	2.18 × 10^−70^	0.777
insomnia	IVW	205	−0.195	0.028	0.823 (0.779–0.869)	2.78 × 10^−12^	4.34 × 10^−38^	0.496
Physical measurements								
Body mass index (BMI)	IVW	205	−0.686	0.065	0.504 (0.443–0.573)	8.18 × 10^−26^	3.08 × 10^−253^	0.373
Body fat percentage	IVW	205	−0.527	0.049	0.591 (0.536–0.651)	1.73 × 10^−26^	8.10 × 10^−234^	0.714
Waist circumference	IVW	205	−0.505	0.054	0.603 (0.543–0.671)	1.12 × 10^−20^	3.70 × 10^−198^	0.374
Hip circumference	IVW	205	−0.379	0.066	0.684 (0.602–0.779)	8.93 × 10^−9^	1.45 × 10^−254^	0.272
Systolic blood pressure	IVW	15	−0.249	0.215	0.780 (0.511–1.188)	2.47 × 10^−1^	3.39 × 10^−33^	0.793
Diastolic blood pressure	IVW	15	−0.268	0.228	0.765 (0.489–1.196)	2.40 × 10^−1^	1.77 × 10^−40^	0.563

**Table 3.** Effects of each mediator between educational attainment (EA) and IgAN.

Mediator	Mediating effects	95%CI	*p*	Proportion Mediated	95%CI
insomnia	−0.099	−0.175, −0.023	0.011	12.522	2.372–22.671
Body mass index (BMI)	−0.087	−0.145, −0.030	0.003	11.056	3.440–18.673
Waist circumference	−0.078	−0.132, −0.024	0.004	9.933	2.785–17.109

## Discussion

In this MR study, we comprehensively analyzed for the first time the causal relationship between 68 environmental factors and IgAN and MN. We identified 20 sets of causal associations, including 8 novel pairs and validated 12 previously reported pairs. This research provides a potential foundation for the establishment of precise etiological and risk assessment evidence for IgAN and MN.

Socioeconomic factors have a significant impact on the development and prognosis of numerous diseases [13]. The evidence linking autoimmune kidney disease to socioeconomic status is limited and inconsistent. A study has shown that greater social deprivation is associated with a higher incidence of IgAN [14]. Conversely, a retrospective cohort study showed no statistically significant association between socioeconomic status and the incidence of IgAN or MN [15]. Additionally, a 6.5-year multicenter observational study even found a negative link between poor education and IgAN [16]. Our findings revealed a negative correlation between EA and the risk of IgAN and MN. Likewise, we noted a negative association between average household economic income and the risk of IgAN, and this association is unidirectional through our MR analysis. Hypothetical reasons for the inconsistencies between prior studies and our findings may include variations in sample sources, disparities in medical care levels, and the impact of potential confounding factors. Furthermore, our mediated MR analyses revealed that EA may protect against IgAN by reducing sleeplessness and lowering BMI and waist circumference. Similarly, these findings have been corroborated in mediated MR analyses of other diseases [10,17,18]. Education influences the type of occupation and consequently, the corresponding economic income [19]. Furthermore, higher levels of education facilitate access to social resources, including enhanced health knowledge and improved health facilities, thereby contributing to disease prevention [20]. In conclusion, socioeconomic disadvantage is a well-established risk factor for many diseases [12], as confirmed by our MR analyses.

The relationship between individual behavioral habits and autoimmune kidney disease is still unclear. Observational studies have previously reported smoking as a risk factor for the incidence and prognosis of IgAN [7]. Utilizing MR analysis, we examined the impact of varying smoking statuses on IgAN and uncovered no causal link. Notably, we observed a positive correlation between frequent alcohol consumption and an elevated risk of IgAN. An animal study conducted in 1990 demonstrated that chronic ethanol consumption can induce IgAN [21]. Another study demonstrated that alcoholic cirrhosis is related to secondary IgAN [22]. Therefore, we hypothesized that the causal relationship between alcohol intake frequency and IgAN could be mediated by liver disease and needs further studies. A number of previous observational studies have reported the relationship between dietary intake and IgAN. In the dietary habits subgroup, our findings indicated that a gluten-free diet, cheese intake, and fresh fruit intake were associated with a lower risk of IgAN. The gluten-free diet reduces the risk of IgAN, which is consistent with the current prevailing opinion [23]. Case reports and observational studies suggest that a gluten-rich diet may contribute to the association between celiac disease, inflammatory bowel disease, and IgAN [7]. Experimental studies have confirmed that gluten proteins can be involved in the common mechanism of inflammatory bowel disease and IgAN by binding to HLA-DQ2 or-DQ8 in the intestinal mucosa [24,25]. Notably, we discovered that cheese consumption reduced the risk of IgAN, which is consistent with an MR study [26]. The risk of cheese and IgAN remains unclear. The main components of cheese are proteins and probiotics. Studies have reported that high protein intake (e.g., red meat, eggs, and milk) may lead to increased intraglomerular pressure and glomerular hyperfiltration, which may result in structural damage to the glomerulus [27]. However, studies have also confirmed the protective effect of lactobacillus on kidney injury [28], supplementation with probiotics such as lactobacillus and bifidobacterium significantly improves gut ecological dysbiosis and provides significant renal protection in IgAN [29,30]. In addition, the antioxidants and minerals it contains might be beneficial for IgAN [26]. Current reports on the association between cheese intake and IgAN are limited, and it would be valuable to conduct future experimental studies to explore the biological plausibility. We also found that fresh fruit intake reduced the risk of IgAN, which is consistent with previous reports that phytonutrients and plant-based diets (e.g., vegetables and fruits) have a beneficial effect on patients with chronic kidney disease [31]. In inverse MR analysis, we found that IgAN was negatively associated with cheese and gluten-free diets, this means that patients with IgAN tend to consume a gluten-rich diet and less cheese. Nevertheless, the limited number of SNPs and the presence of reverse causal correlations may have introduced bias into our conclusions. Furthermore, we also found that beef intake was associated with MN risk. However, the link with extreme odds ratios (OR = 56.109), which does not seem reasonable. We assume that the fewer IVs may contribute to these extreme odds ratios. The link was supported by the weighted median, leave-one-out method in sensitivity analyses. Research has found that cationic bovine serum albumin leads to early-childhood membranous nephropathy through binding to the anionic glomerular capillary wall and *in situ* formation of immune complexes [32]. Despite the interference of unmeasured confounders, the existence of the association cannot be totally denied. Additional experimental investigations are required to elucidate the role of different food antigens in IgAN and MN. Our study is the first to identify a correlation between insomnia and an elevated risk of IgAN, inflammation may play a role in the link between sleep problems and autoimmune illnesses [33]. The specific biological mechanisms are speculative, which suggests new directions for future research. Regarding physical activity, our research reveals for the first time that engaging in moderate to vigorous physical activity is associated with a reduced risk of MN. Previous research has indicated that exercise appears to have a dose-dependent relationship with health outcomes and inflammatory mediators [34]. Current knowledge regarding the associations between sleep, exercise, and glomerular diseases is scarce, necessitating prospective studies with larger sample sizes for validation. Consequently, ensuring adequate sleep and engaging in regular exercise are viable strategies for disease prevention.

In lipid metabolism, we found that triglycerides (TG) were associated with a higher risk of IgAN. An MR analysis also verified the causal relationship between TG and IgAN [35]. The podocyte apoptosis and endothelial dysfunction due to hypertriglyceridemia are possible explanations [35]. We used the most recent GWAS data to conduct reverse MR and discovered that IgAN promotes the elevation of TG. This suggests that the relationship between IgAN and triglycerides is bidirectional. Studies have shown that lipid metabolism is frequently impaired in patients with chronic kidney disease. Large amounts of proteinuria can lead to hypertriglyceridemia, which is mainly due to impaired clearance and increased biosynthesis of lipoproteins [36]. There is limited clinical evidence that IgAN causes hypertriglyceridemia, and needs further exploration. Among the iron metabolism indicators, we discovered for the first time a link between transferrin saturation and increased IgAN risk. Iron metabolism has been reported to be closely related to the pathogenesis of IgAN [37]. Hamoglobin in hematuria causes iron-dependent oxidative stress, resulting in renal damage as a potential mechanism [38]. Our findings provide evidence to explore the role of iron metabolism in IgAN.

Regarding physical examination indicators, our MR analyses revealed a positive correlation between higher waist circumference, BMI, and body fat percentage, and an increased risk of IgAN. Current evidence from observational studies indicate that BMI is associated with IgAN progression or poor prognosis [39,40]. Obesity contributes to kidney damage potentially through adipocyte-associated chronic, low-grade inflammation as a possible mechanism [41]. However, our MR analyses could not demonstrate that hip circumference was positively associated with the risk of IgAN. Although we used the MR-PRESSO method to exclude outlier SNPs, we were unable to completely eliminate the effect of horizontal pleiotropy, implying that the causal association between hip circumference and IgAN was influenced by confounding factors. Large samples of observational data could be used for future analysis. We also discovered that WHR was a risk factor for MN on an adjusted BMI basis, whereas BMI was not linked with the risk of MN. Chen et al. found that high BMI was associated with mesangial cell proliferation (MCP) and a combination score in IMN patients [42]. However, a study has also found that waist-to-hip ratio, rather than BMI, is related with CKD [43]. The influence of obesity on primary glomerular disease is confusing. Current research has predominantly focused on the effects of BMI, with limited clinical investigations employing waist circumference, hip circumference, WHR, and percentage body fat as metrics to assess the influence of obesity on IgAN and MN. This may be because these indicators are difficult to collect in clinical practice, and our MR analysis overcomes this limitation. Regarding blood pressure, our study revealed that both systolic and diastolic blood pressure were associated with the risk of IgAN, which is consistent with previous MR analyses [44]. Consequently, managing blood pressure serves as a crucial preventive measure against IgAN. Regarding cognitive performance and intelligence, we found that a better cognitive and intellectual level was a protective factor for IgAN, which is consistent with the MR analyses of cognitive and intellectual level in the other diseases, and this association is most likely mediated by socioeconomic and lifestyle factors [11]. However, in the reverse MR analysis, IgAN was found to be negatively correlated with cognitive and intelligence levels. There are no current studies reporting this bidirectional causal association, which provides direction for future research.

In recent years, several studies have reported the association of air pollutants with IgAN and MN. A retrospective study in China found that high levels of PM 2.5, NO, NO_2_, and SO_2_ associated with an increased risk of nephrotic syndrome [45]. The present study demonstrated a causal association between NO exposure and a higher risk of MN. Nitrogen oxide air pollutants stimulate the production of nitrated proteins, potentially triggering autoimmunity [45]. In addition, a retrospective study covering 284 cities in China over a period of 11 years and including a total of 75,163 cases indicated that in areas with PM2.5 concentrations above 70 μg/m^3^, the risk of MN increased by about 14% for every 10 μg/m^3^ increase in PM2.5 concentrations [46]. However, our MR analysis did not reveal a causal relationship between PM2.5 and MN or IgAN, possibly due to the European population used. More study on the geographical differences in PM2.5-MN correlations is required in the future.

Our analysis is subject to several inevitable limitations. First, potentially disease-related exposure factors reported in observational studies, such as silica and mercury, could not be assessed due to restrictions in GWAS data availability. Second, the prevalence of IgAN is significantly higher in East Asian populations. Due to GWAS data restrictions, the study was limited to Europeans, which helped to eliminate demographic information bias but reduced the applicability and relevance of the findings [47]. Third, the presence of heterogeneity was observed in some associations, presumably related to different GWAS analysis platforms, experimental methods and population specificity. For this, we used a random-effects IVW model as a main effect to reduce the effect of heterogeneity, and a leave-one-out analysis to observe changes in heterogeneity. Regrettably, the existing GWAS data do not allow for stratified analysis to better investigate the impact of environmental factors in specific populations. Our research also has some advantages. On the one hand, the impact of various environmental exposures on IgAN and MN was confirmed in an economic and scientific approach using the most latest GWAS data. On the other hand, our more comprehensive analysis provides a foundation for future investigations into the biological mechanisms underpinning the diverse impacts of environmental factors on IgAN and MN.

## Conclusion

Our study provides causality-oriented evidence between 20 genetically predictable environmental factors and the risk of IgAN and MN, which may open new opportunities for developing preventive strategies.

## Supplementary Material

Supplementary material picture 25.tif

Supplementary material picture 11.tif

Supplementary material picture 8.tif

File 6.xlsx

File 5.xlsx

Supplementary material picture 4.tif

Supplementary material picture 3.tif

Supplementary material picture 35.tif

Supplementary material picture 19.tif

Supplementary material picture 12.tif

Supplementary material picture 14.tif

Supplementary material picture 40.tif

Supplementary material picture 26.tif

File 4.xlsx

File 7.docx

Supplementary material picture 21.tif

Supplementary material picture 20.tif

File 1.docx

Supplementary material picture 17.tif

Supplementary material picture 32.tif

File 3.xlsx

Supplementary material picture 24.tif

Supplementary material picture 27.tif

Supplementary material picture 6.tif

Supplementary material picture 23.tif

Supplementary material picture 5.tif

Supplementary material picture 44.tif

Supplementary material picture 13.tif

Supplementary material picture 37.tif

File 2.xlsx

Supplementary material picture 34.tif

Supplementary material picture 33.tif

Supplementary material picture 28.tif

Supplementary material picture 15.tif

Supplementary material picture 43.tif

Supplementary material picture 9.tif

Supplementary material picture 1.tif

Supplementary material picture 39.tif

Supplementary material picture 41.tif

Supplementary material picture 10.tif

Supplementary material picture 16.tif

Supplementary material picture 30.tif

Supplementary material picture 18.tif

Supplementary material picture 2.tif

Supplementary material picture 22.tif

Supplementary material picture 36.tif

Supplementary material picture 7.tif

Supplementary material picture 38.tif

Supplementary material picture 29.tif

Supplementary material picture 42.tif

Supplementary material picture 31.tif

List of supplementary material.docx

## Data Availability

All the datasets were derived from sources in the public domain: GWAS catalog and IEU OpenGWAS project. Supplementary material can be obtained by contacting the author.

## References

[1] Yang C, Gao B, Zhao X, et al. Executive summary for China kidney disease network (CK-NET) 2016 annual data report. Kidney Int. 2020;98(6):1419–1423. doi: 10.1016/j.kint.2020.09.003. 33276868

[2] Lee M, Suzuki H, Nihei Y, et al. Ethnicity and IgA ­nephropathy: worldwide differences in epidemiology, timing of diagnosis, clinical manifestations, management and prognosis. Clin Kidney J. 2023;16(Suppl 2):ii1–ii8. doi: 10.1093/ckj/sfad199. 38053973 PMC10695519

[3] Stamellou E, Seikrit C, Tang SCW, et al. IgA nephropathy. Nat Rev Dis Primers. 2023;9(1):67. doi: 10.1038/s41572-023-00476-9. 38036542

[4] Ronco P, Beck L, Debiec H, et al. Membranous nephropathy. Nat Rev Dis Primers. 2021;7(1):69. doi: 10.1038/s41572-021-00303-z. 34593809

[5] Claudio P. Primary membranous nephropathy: an endless story. J Nephrol. 2023;36(2):563–574. Epub 2022 Oct 17. PMID: 36251213. doi: 10.1007/s40620-022-01461-3. 36251213

[6] Fan P, Song J, Chen Q, et al. The influence of ­environmental factors on clinical pathological changes of patients with immunoglobulin A nephropathy from different areas of China. Ren Fail. 2018;40(1):597–602. doi: 10.1080/0886022X.2018.1532907. 30373437 PMC6211320

[7] Xia J, Wang M, Jiang W. New insights into pathogenesis of IgA nephropathy. Int Urol Nephrol. 2022;54(8):1873–1880. doi: 10.1007/s11255-021-03094-0. 35048307

[8] Zhang XD, Cui Z, Zhao MH. The genetic and environmental factors of primary membranous nephropathy: an overview from China. Kidney Dis. 2018;4(2):65–73. doi: 10.1159/000487136. PMC6029227 29998121

[9] Larsson SC, Butterworth AS, Burgess S. Mendelian randomization for cardiovascular diseases: principles and applications. Eur Heart J. 2023;44(47):4913–4924. doi: 10.1093/eurheartj/ehad736. 37935836 PMC10719501

[10] Lan G, Xie M, Lan J, et al. Association and mediation between educational attainment and respiratory diseases: a Mendelian randomization study. Respir Res. 2024;25(1):115. doi: 10.1186/s12931-024-02722-4. 38448970 PMC10918882

[11] Wang Y, Ye C, Kong L, et al. Independent associations of ­education, intelligence, and cognition with hypertension and the mediating effects of cardiometabolic risk factors: a Mendelian randomization study. Hypertension. 2023;80(1):192–203. doi: 10.1161/HYPERTENSIONAHA.122.20286. 36353998 PMC9722390

[12] Xie J, Liu L, Mladkova N, et al. The genetic architecture of membranous nephropathy and its potential to ­improve non-invasive diagnosis. Nat Commun. 2020;11(1):1600. doi: 10.1038/s41467-020-15383-w. 32231244 PMC7105485

[13] Ye CJ, Liu D, Chen ML, et al. Mendelian randomization evidence for the causal effect of mental well-being on healthy aging. Nat Hum Behav. 2024;8(9):1798–1809. doi: 10.1038/s41562-024-01905-9. 38886532

[14] Mcquarrie EP, Mackinnon B, Mcneice V, et al. The incidence of biopsy-proven IgA nephropathy is associated with multiple socioeconomic deprivation. Kidney Int. 2014;85(1):198–203. doi: 10.1038/ki.2013.329. 24025641

[15] Canney M, Induruwage D, Sahota A, et al. Socioeconomic position and incidence of glomerular diseases. Clin J Am Soc Nephrol. 2020;15(3):367–374. doi: 10.2215/CJN.08060719. 32079609 PMC7057310

[16] Winitzki D, Zacharias HU, Nadal J, et al. Educational attainment is associated with kidney and cardiovascular outcomes in the German CKD (GCKD) cohort. Kidney Int Rep. 2022;7(5):1004–1015. doi: 10.1016/j.ekir.2022.02.001. 35570994 PMC9091575

[17] Rogne T, Gill D, Liew Z, et al. Mediating factors in the association of maternal educational level with pregnancy outcomes: a mendelian randomization study. JAMA Netw Open. 2024;7(1):e2351166. doi: 10.1001/jamanetworkopen.2023.51166. 38206626 PMC10784860

[18] Zhang J, Chen Z, Pärna K, et al. Mediators of the ­association between educational attainment and type 2 ­diabetes mellitus: a two-step multivariable Mendelian randomisation study. Diabetologia. 2022;65(8):1364–1374. doi: 10.1007/s00125-022-05705-6. 35482055 PMC9283137

[19] Glei DA, Lee C, Weinstein M. Assessment of mortality disparities by wealth relative to other measures of socioeconomic status among US adults. JAMA Netw Open. 2022;5(4):e226547. doi: 10.1001/jamanetworkopen.2022.6547. 35394513 PMC8994125

[20] Zajacova A, Lawrence EM. The relationship between education and health: reducing disparities through a contextual approach. Annu Rev Public Health. 2018;39(1):273–289. Epub 2018 Jan 12. PMID: 29328865; PMCID: PMC5880718. doi: 10.1146/annurev-publhealth-031816-044628. 29328865 PMC5880718

[21] Smith SM, Yu GS, Tsukamoto H. IgA nephropathy in ­alcohol abuse. An animal model. Lab Invest. 1990;62(2):179–184. PMID: 2304330. 2304330

[22] Novak J, Julian BA. Sugars and alcohol: igA-associated renal diseases in alcoholic cirrhosis. Kidney Int. 2011;80(12):1252–1254. doi: 10.1038/ki.2011.302. 22126980

[23] Cheung CK, Barratt J. Gluten and IgA nephropathy: you are what you eat? Kidney Int. 2015;88(2):215–218. doi: 10.1038/ki.2015.149. 26230197

[24] Collin P, Syrjänen J, Partanen J, et al. Celiac disease and HLA DQ in patients with IgA nephropathy. Am J Gastroenterol. 2002;97(10):2572–2576. doi: 10.1111/j.1572-0241.2002.06025.x. 12385441

[25] Papista C, Lechner S, Ben Mkaddem S, et al. Gluten exacerbates IgA nephropathy in humanized mice through gliadin-CD89 interaction. Kidney Int. 2015;88(2):276–285. doi: 10.1038/ki.2015.94. 25807036

[26] Li Y, Wan S, Liu J, et al. Causal relationship between dietary intake and IgA nephropathy: a Mendelian randomization study. Front Nutr. 2024;11:1400907. doi: 10.3389/fnut.2024.1400907. 39285865 PMC11403370

[27] Ko GJ, Obi Y, Tortorici AR, et al. Dietary protein intake and chronic kidney disease. Curr Opin Clin Nutr Metab Care. 2017;20(1):77–85. doi: 10.1097/MCO.0000000000000342. 27801685 PMC5962279

[28] Kim H, Nam BY, Park J, et al. Lactobacillus acidophilus KBL409 reduces kidney fibrosis via immune modulatory effects in mice with chronic kidney disease. Mol Nutr Food Res. 2022;66(22):e2101105. doi: 10.1002/mnfr.202101105. 36059191

[29] Tan J, Dong L, Jiang Z, et al. Probiotics ameliorate IgA nephropathy by improving gut dysbiosis and blunting NLRP3 signaling. J Transl Med. 2022;20(1):382. doi: 10.1186/s12967-022-03585-3. 36038927 PMC9422169

[30] Zhu H, Cao C, Wu Z, et al. The probiotic *L. casei* Zhang slows the progression of acute and chronic kidney disease. Cell Metab. 2021;33(10):1926–1942.e8. doi: 10.1016/j.cmet.2021.06.014. 34270930

[31] Carrero JJ, González-Ortiz A, Avesani CM, et al. Plant-based diets to manage the risks and complications of chronic kidney disease. Nat Rev Nephrol. 2020;16(9):525–542. doi: 10.1038/s41581-020-0297-2. 32528189

[32] Debiec H, Lefeu F, Kemper MJ, et al. Early-childhood membranous nephropathy due to cationic bovine serum albumin. N Engl J Med. 2011;364(22):2101–2110. doi: 10.1056/NEJMoa1013792. 21631322

[33] Zielinski MR, Systrom DM, Rose NR. Fatigue, sleep, and autoimmune and related disorders. Front Immunol. 2019;10:1827. doi: 10.3389/fimmu.2019.01827. 31447842 PMC6691096

[34] Metsios GS, Moe RH, Kitas GD. Exercise and inflammation. Best Pract Res Clin Rheumatol. 2020;34(2):101504. doi: 10.1016/j.berh.2020.101504. 32249021

[35] Yang Y, Li Y, Feng X, et al. The causal effect of triglyceride and high blood pressure on IgA nephropathy: a Mendelian randomization study. Front Med (Lausanne). 2024;11:1338462. doi: 10.3389/fmed.2024.1338462. 38390575 PMC10881685

[36] Agrawal S, Zaritsky JJ, Fornoni A, et al. Dyslipidaemia in nephrotic syndrome: mechanisms and treatment. Nat Rev Nephrol. 2018;14(1):57–70. doi: 10.1038/nrneph.2017.155. 29176657 PMC5770189

[37] Tian ZY, Li Z, Chu L, et al. Iron metabolism and chronic inflammation in IgA nephropathy. Ren Fail. 2023;45(1):2195012. doi: 10.1080/0886022X.2023.2195012. 37013479 PMC10075521

[38] Nath KA. Tubulointerstitial changes as a major determinant in the progression of renal damage. Am J Kidney Dis. 1992;20(1):1–17. doi: 10.1016/s0272-6386(12)80312-x. 1621674

[39] Ariyasu Y, Torikoshi K, Tsukamoto T, et al. Analysis of the impact of obesity on the prognosis of IgA nephropathy according to renal function and sex. Clin Exp Nephrol. 2024;28(11):1155–1167. doi: 10.1007/s10157-024-02519-1. 38831156

[40] Bonnet F, Deprele C, Sassolas A, et al. Excessive body weight as a new independent risk factor for clinical and pathological progression in primary IgA nephritis. Am J Kidney Dis. 2001;37(4):720–727. doi: 10.1016/s0272-6386(01)80120-7. 11273871

[41] Shimamoto M, Ohsawa I, Suzuki H, et al. Impact of body mass index on progression of IgA nephropathy among Japanese patients. J Clin Lab Anal. 2015;29(5):353–360. doi: 10.1002/jcla.21778. 25131157 PMC6807233

[42] Chen X, Chen S, Li Z, et al. Correlation of body mass index with clinicopathologic parameters in patients with idiopathic membranous nephropathy. Diabetes Metab Syndr Obes. 2022;15:1897–1909. doi: 10.2147/DMSO.S366100. 35757192 PMC9231685

[43] Ouyang Y, Xie J, Yang M, et al. Underweight is an independent risk factor for renal function deterioration in patients with IgA nephropathy. PLoS One. 2016;11(9):e0162044. doi: 10.1371/journal.pone.0162044. 27611091 PMC5017745

[44] Liu B, Zhao L, Yang Q, et al. Hyperuricemia and hypertriglyceridemia indicate tubular atrophy/interstitial fibrosis in patients with IgA nephropathy and membranous nephropathy. Int Urol Nephrol. 2021;53(11):2321–2332. doi: 10.1007/s11255-021-02844-4. 33895976

[45] Lin SY, Hsu WH, Lin CL, et al. Association of exposure to fine-particulate air pollution and acidic gases with incidence of nephrotic syndrome. Int J Environ Res Public Health. 2018;15(12):2860. doi: 10.3390/ijerph15122860. 30558173 PMC6313436

[46] Xu X, Wang G, Chen N, et al. Long-term exposure to air pollution and increased risk of membranous nephropathy in China. J Am Soc Nephrol. 2016;27(12):3739–3746. doi: 10.1681/ASN.2016010093. 27365535 PMC5118492

[47] Qing J, Li Y, Soliman KM, et al. A practical guide for nephrologist peer reviewers: understanding and appraising Mendelian randomization studies. Ren Fail. 2025;47(1):2445763. doi: 10.1080/0886022X.2024.2445763. 39806780 PMC11734392

