# Supplementary figures and images for: Causal associations between environmental factors and risk of IgA nephropathy and membranous nephropathy: a bidirectional Mendelian randomization and mediation analysis
Source: Ren Fail. 2025 Apr 9;47(1):2486620. doi: 10.1080/0886022X.2025.2486620 (PMC11983537; doi:10.1080/0886022X.2025.2486620)

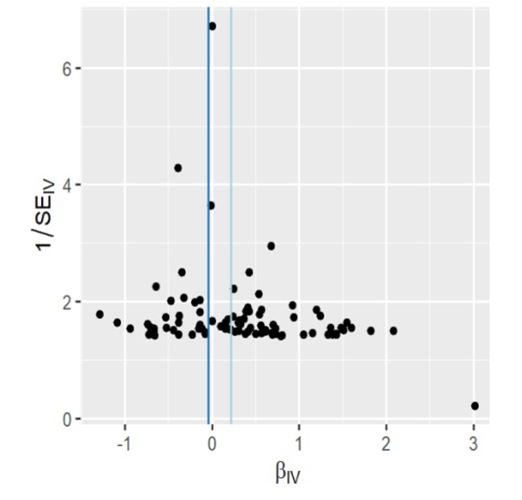

Supplement: Supplementary material picture 25.tif [file IRNF_A_2486620_SM8339.tif]

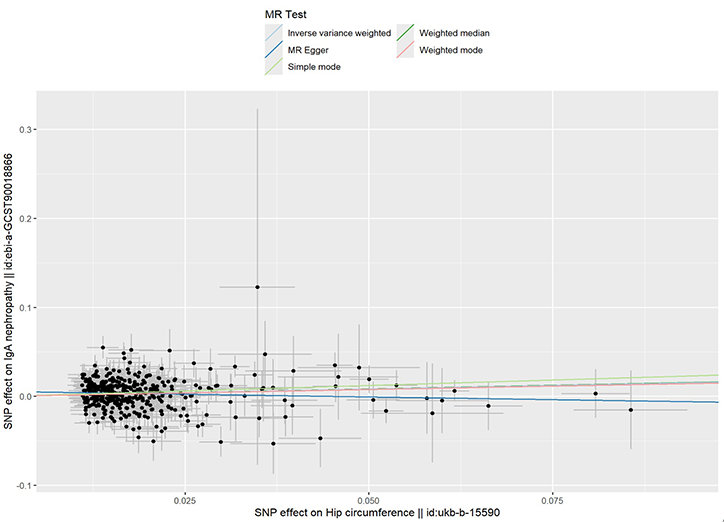

Supplement: Supplementary material picture 11.tif [file IRNF_A_2486620_SM8338.tif]

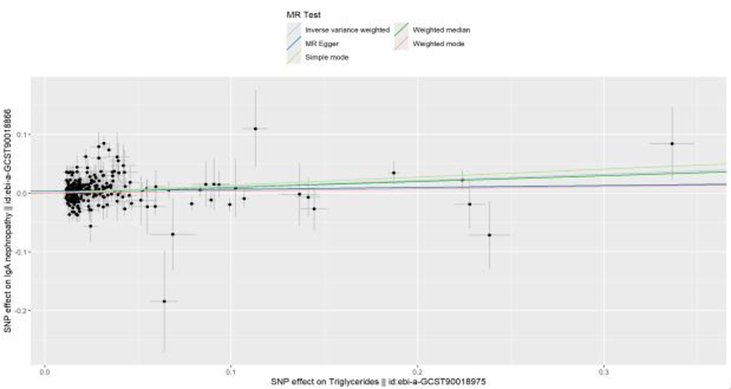

Supplement: Supplementary material picture 8.tif [file IRNF_A_2486620_SM8337.tif]

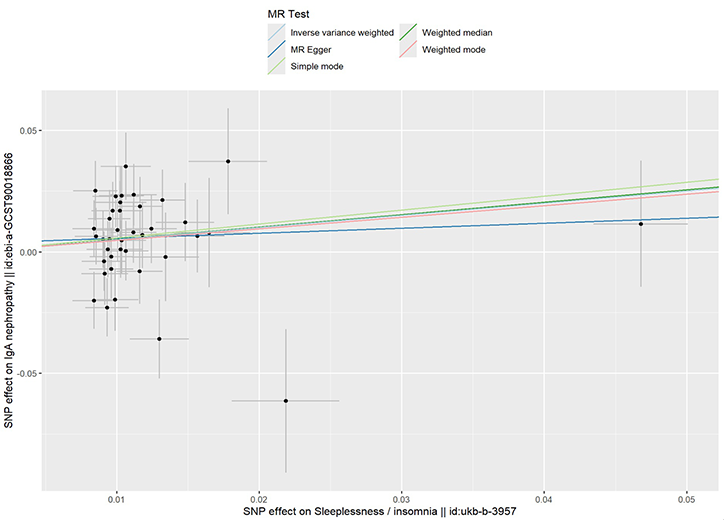

Supplement: Supplementary material picture 4.tif [file IRNF_A_2486620_SM8334.tif]

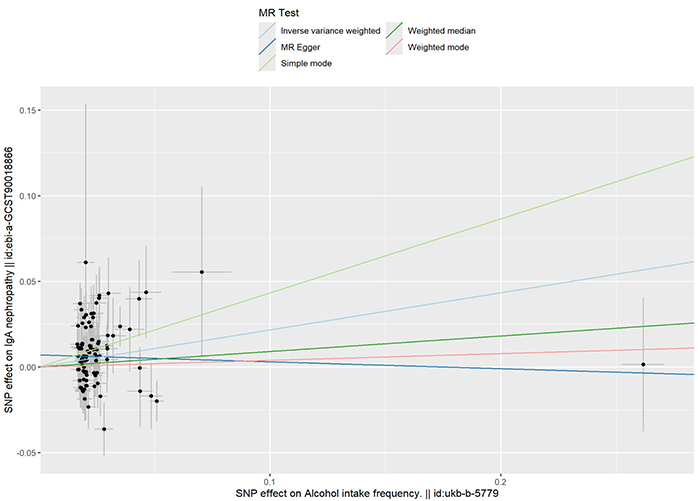

Supplement: Supplementary material picture 3.tif [file IRNF_A_2486620_SM8333.tif]

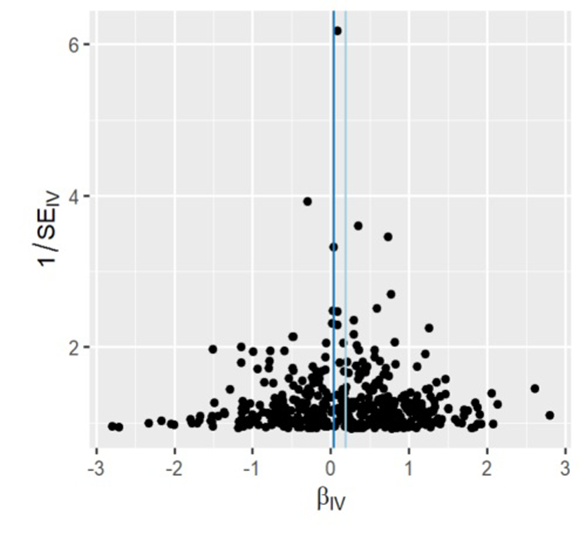

Supplement: Supplementary material picture 35.tif [file IRNF_A_2486620_SM8332.tif]

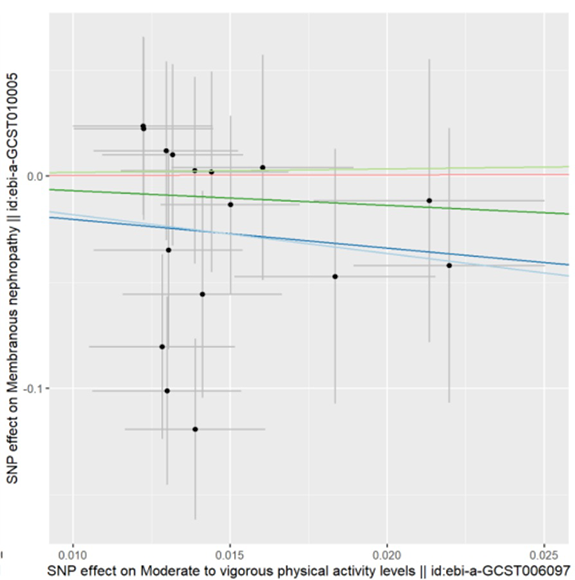

Supplement: Supplementary material picture 19.tif [file IRNF_A_2486620_SM8331.tif]

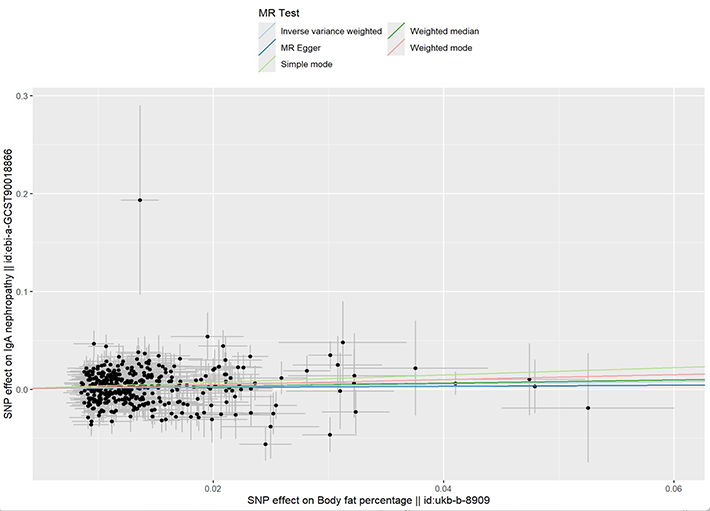

Supplement: Supplementary material picture 12.tif [file IRNF_A_2486620_SM8330.tif]

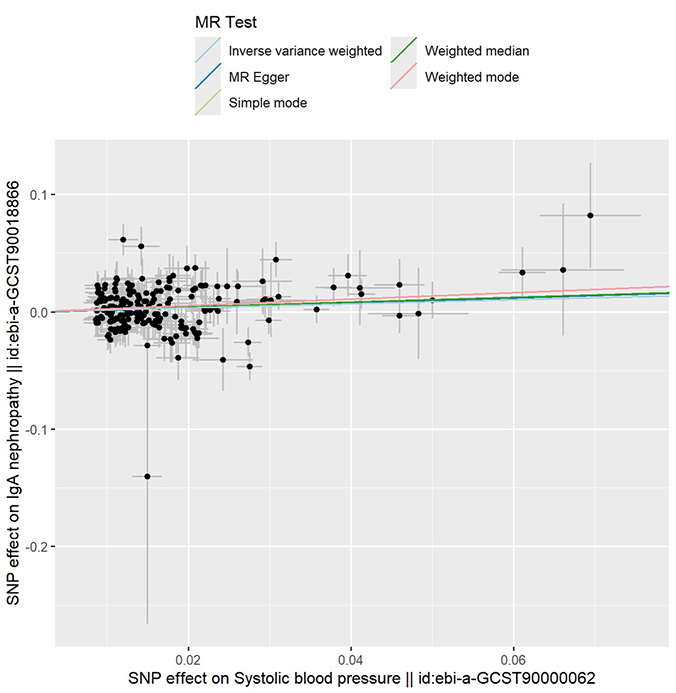

Supplement: Supplementary material picture 14.tif [file IRNF_A_2486620_SM8329.tif]

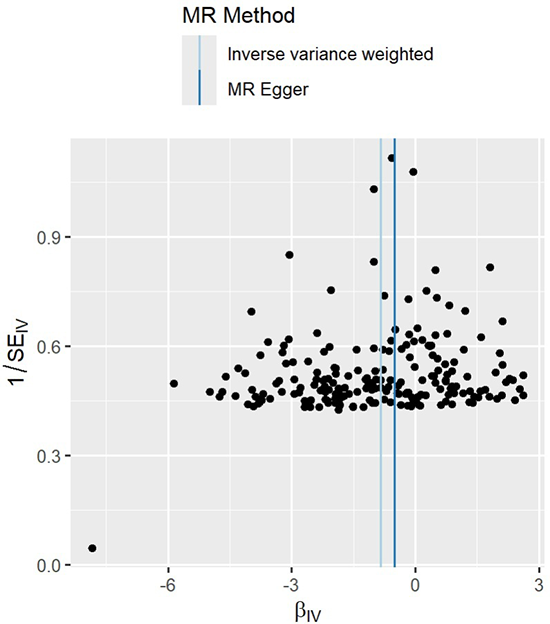

Supplement: Supplementary material picture 40.tif [file IRNF_A_2486620_SM8328.tif]

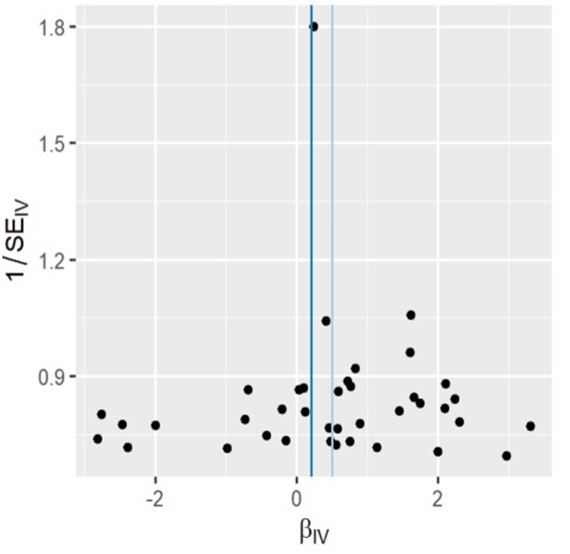

Supplement: Supplementary material picture 26.tif [file IRNF_A_2486620_SM8327.tif]

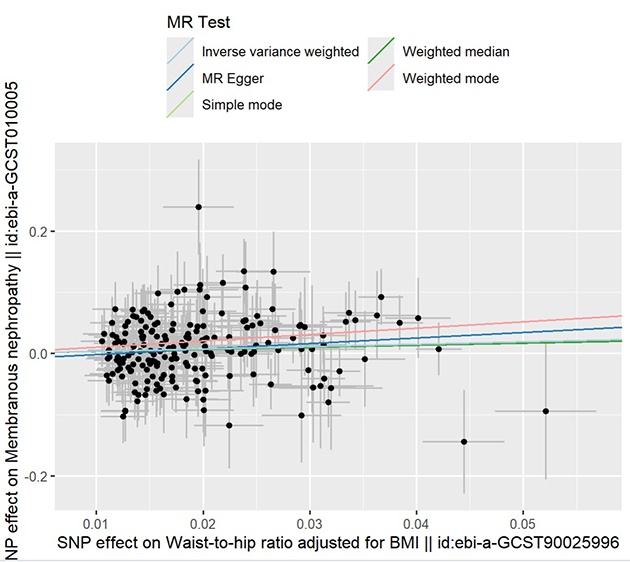

Supplement: Supplementary material picture 21.tif [file IRNF_A_2486620_SM8324.tif]

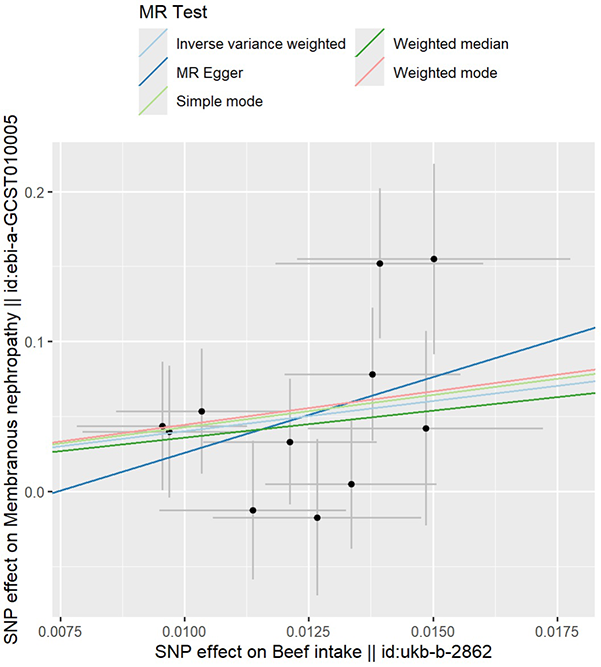

Supplement: Supplementary material picture 20.tif [file IRNF_A_2486620_SM8323.tif]

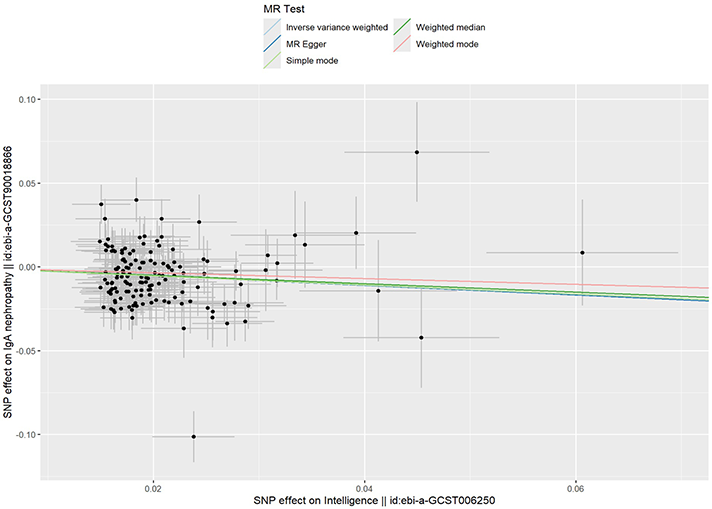

Supplement: Supplementary material picture 17.tif [file IRNF_A_2486620_SM8321.tif]

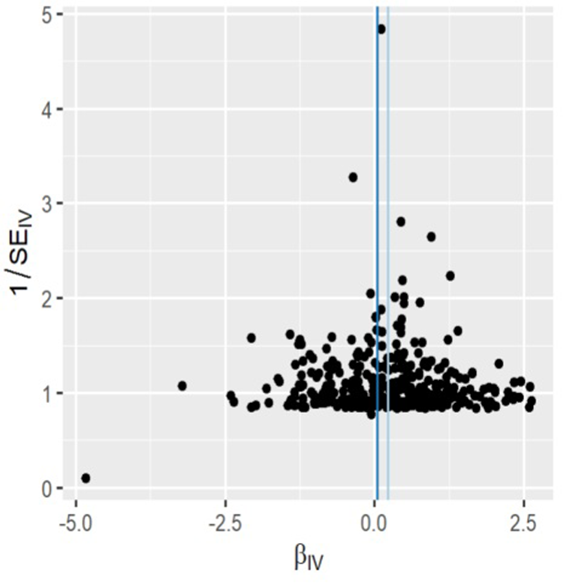

Supplement: Supplementary material picture 32.tif [file IRNF_A_2486620_SM8320.tif]

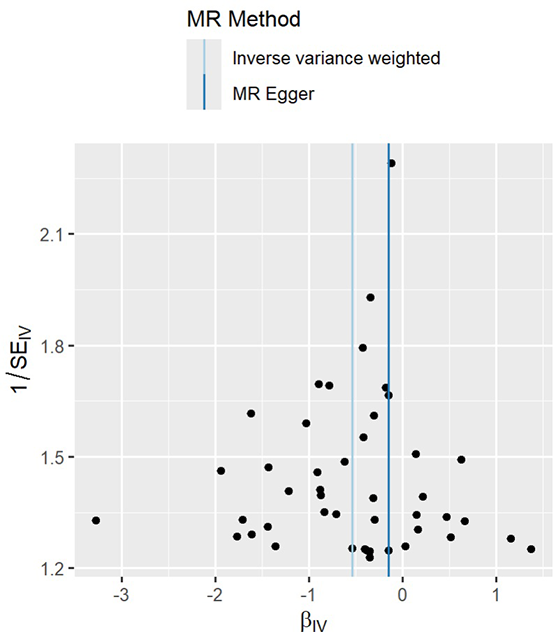

Supplement: Supplementary material picture 24.tif [file IRNF_A_2486620_SM8318.tif]

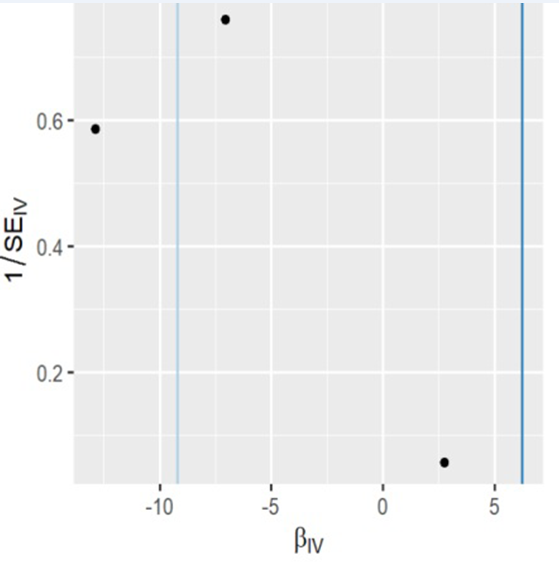

Supplement: Supplementary material picture 27.tif [file IRNF_A_2486620_SM8317.tif]

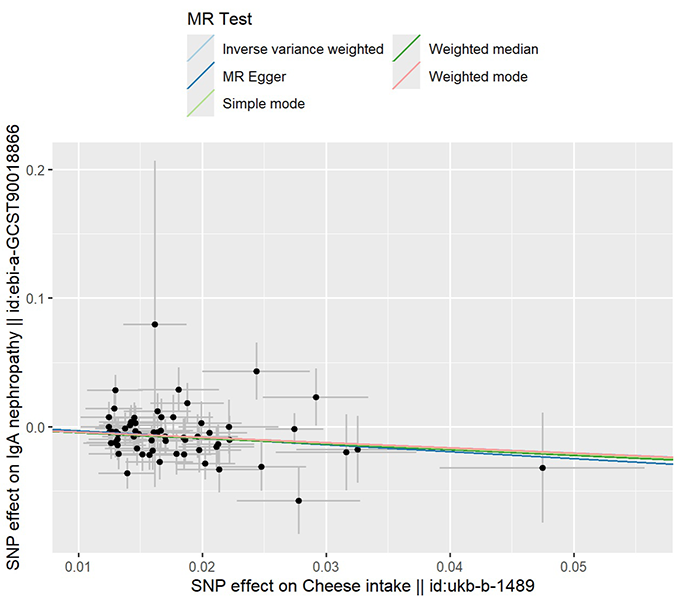

Supplement: Supplementary material picture 6.tif [file IRNF_A_2486620_SM8316.tif]

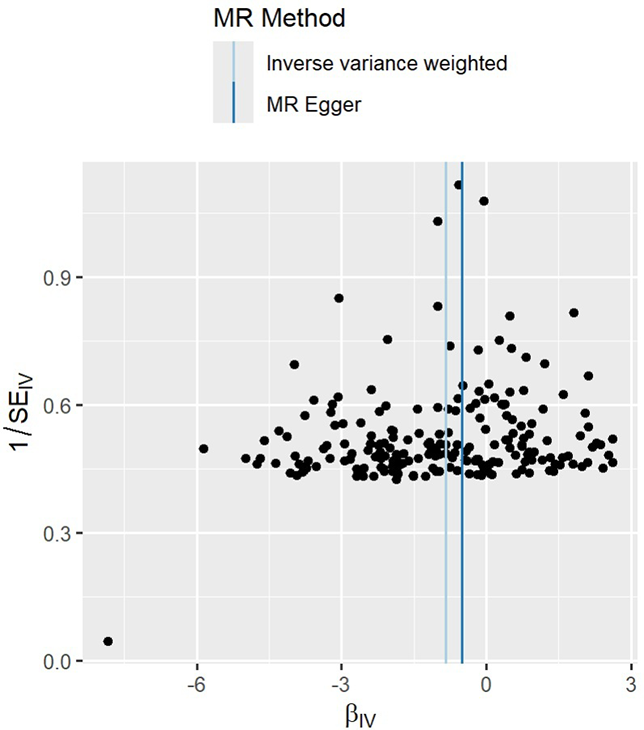

Supplement: Supplementary material picture 23.tif [file IRNF_A_2486620_SM8315.tif]

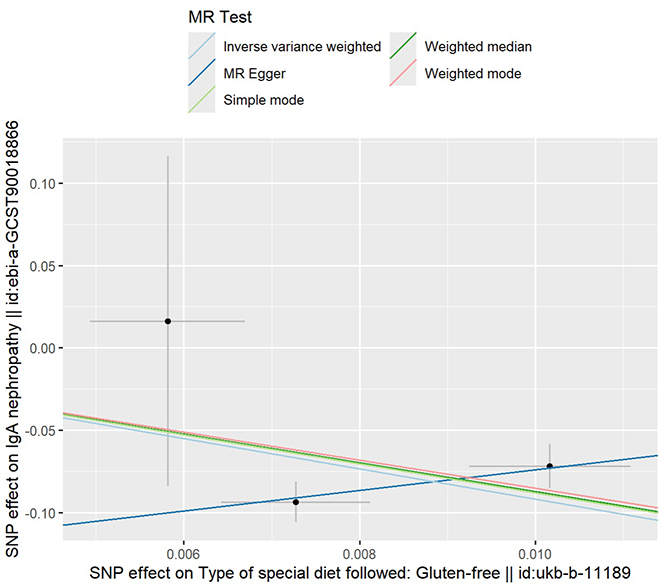

Supplement: Supplementary material picture 5.tif [file IRNF_A_2486620_SM8314.tif]

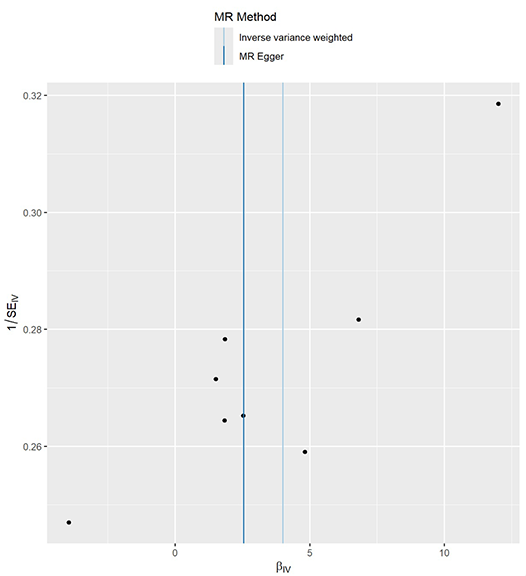

Supplement: Supplementary material picture 44.tif [file IRNF_A_2486620_SM8313.tif]

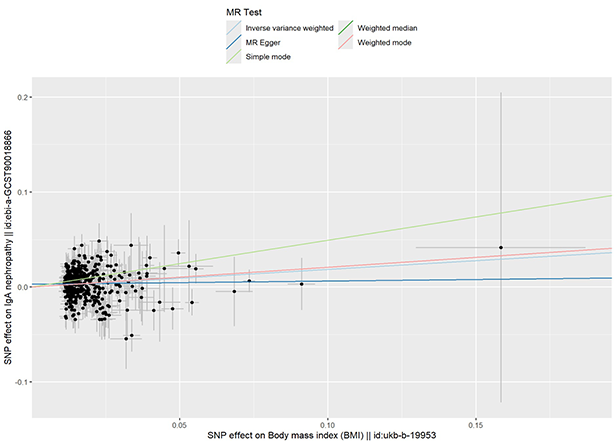

Supplement: Supplementary material picture 13.tif [file IRNF_A_2486620_SM8312.tif]

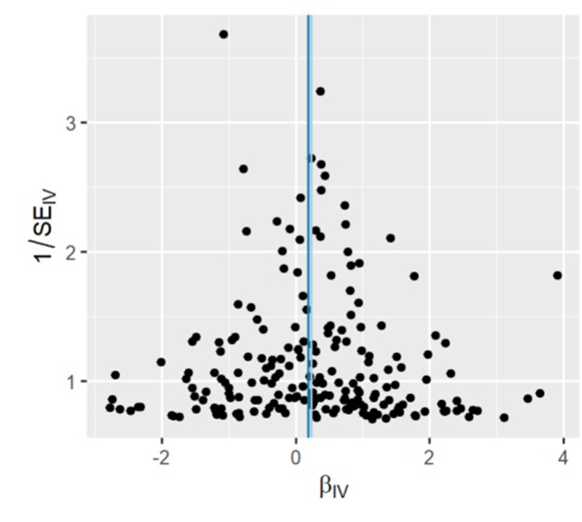

Supplement: Supplementary material picture 37.tif [file IRNF_A_2486620_SM8311.tif]

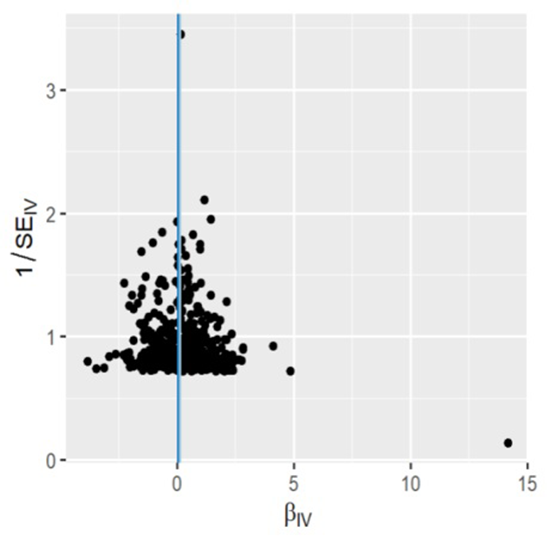

Supplement: Supplementary material picture 34.tif [file IRNF_A_2486620_SM8309.tif]

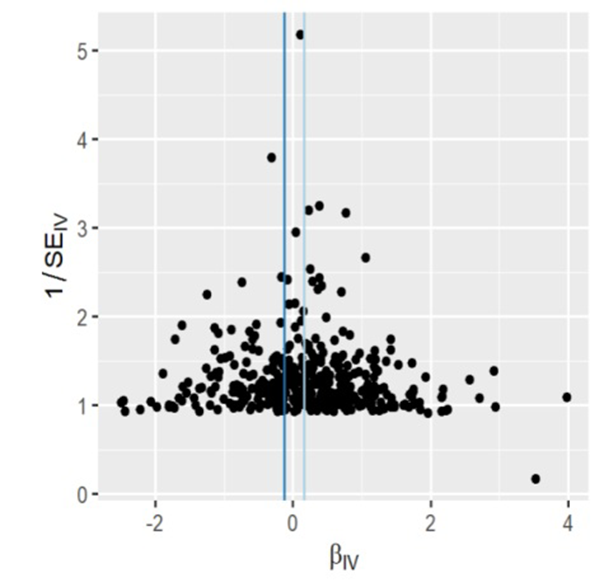

Supplement: Supplementary material picture 33.tif [file IRNF_A_2486620_SM8308.tif]

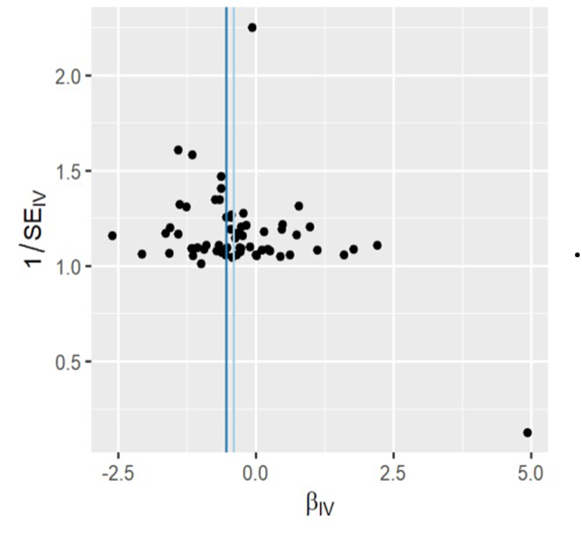

Supplement: Supplementary material picture 28.tif [file IRNF_A_2486620_SM8307.tif]

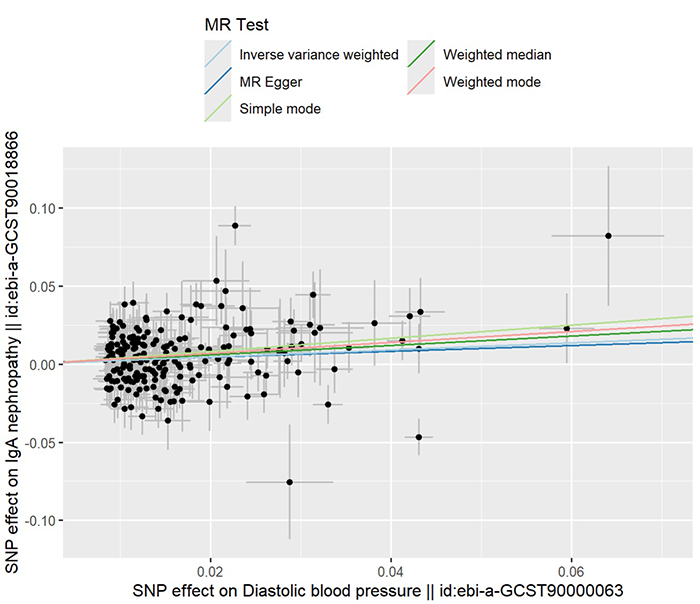

Supplement: Supplementary material picture 15.tif [file IRNF_A_2486620_SM8306.tif]

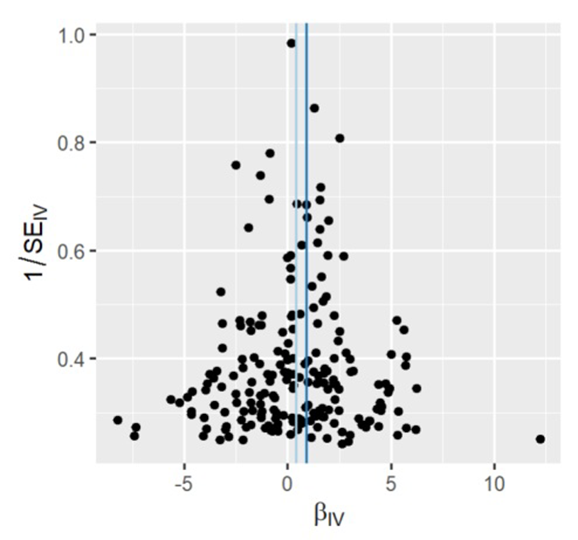

Supplement: Supplementary material picture 43.tif [file IRNF_A_2486620_SM8305.tif]

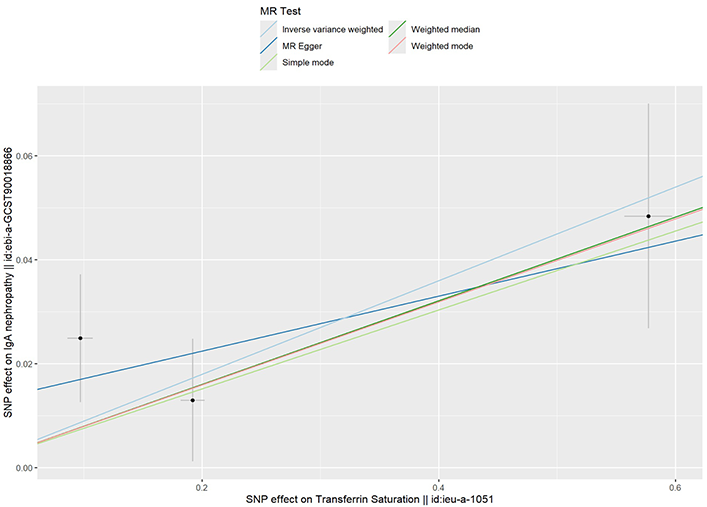

Supplement: Supplementary material picture 9.tif [file IRNF_A_2486620_SM8304.tif]

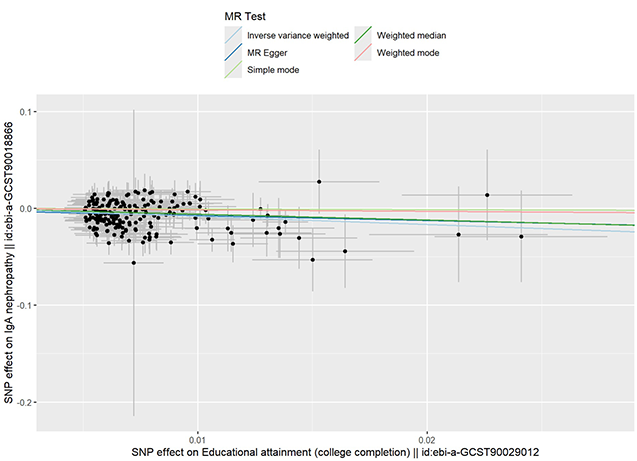

Supplement: Supplementary material picture 1.tif [file IRNF_A_2486620_SM8303.tif]

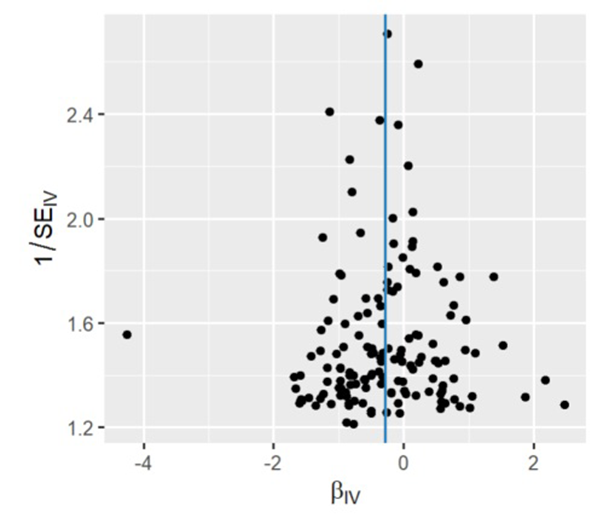

Supplement: Supplementary material picture 39.tif [file IRNF_A_2486620_SM8302.tif]

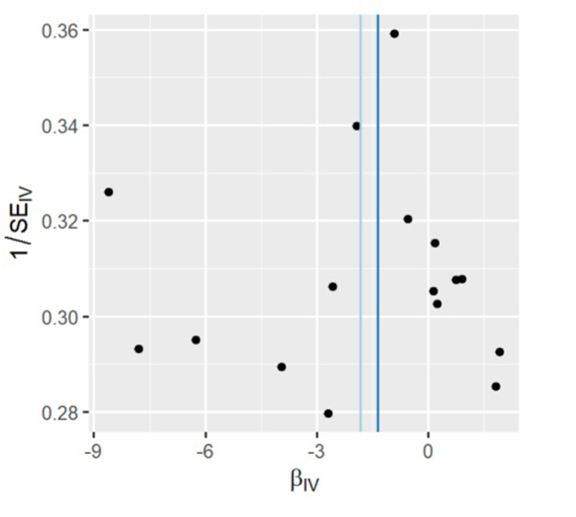

Supplement: Supplementary material picture 41.tif [file IRNF_A_2486620_SM8301.tif]

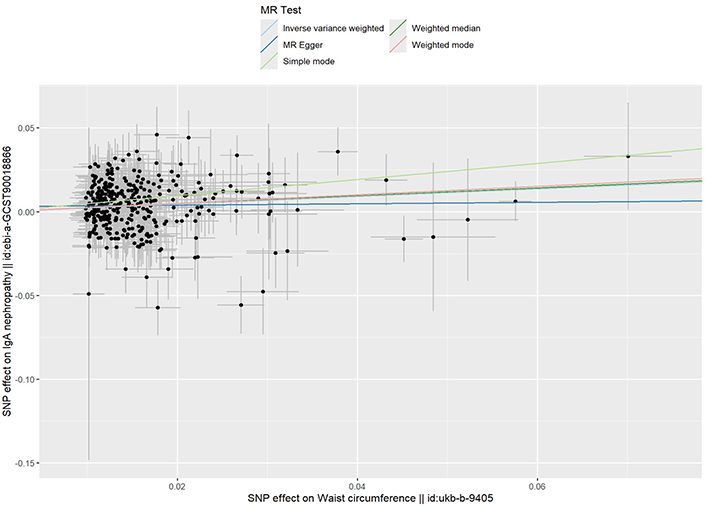

Supplement: Supplementary material picture 10.tif [file IRNF_A_2486620_SM8300.tif]

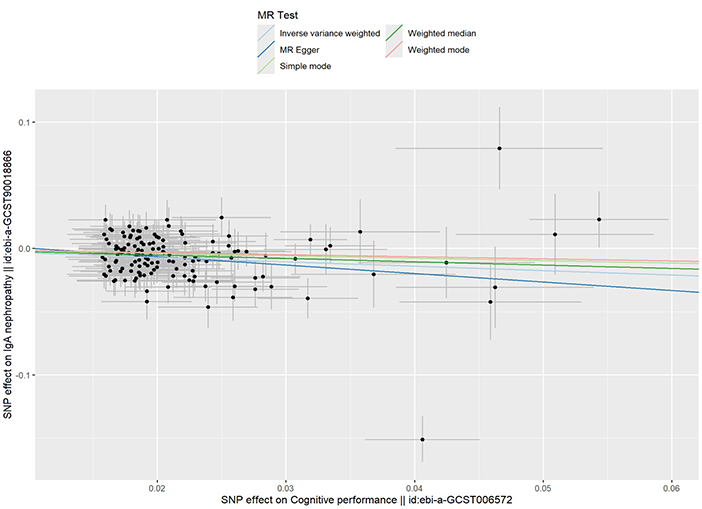

Supplement: Supplementary material picture 16.tif [file IRNF_A_2486620_SM8299.tif]

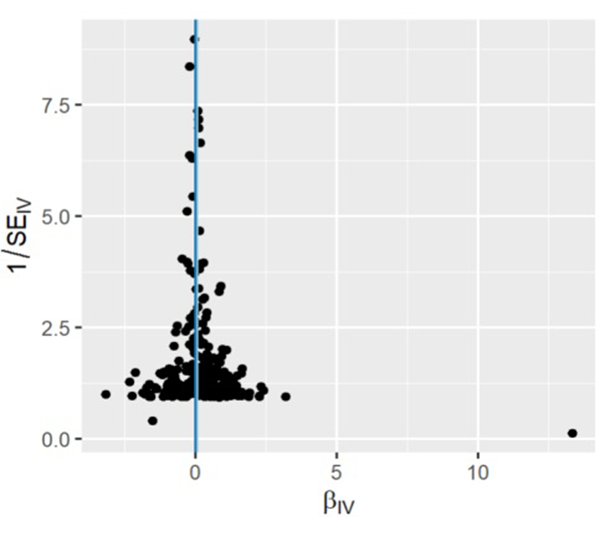

Supplement: Supplementary material picture 30.tif [file IRNF_A_2486620_SM8298.tif]

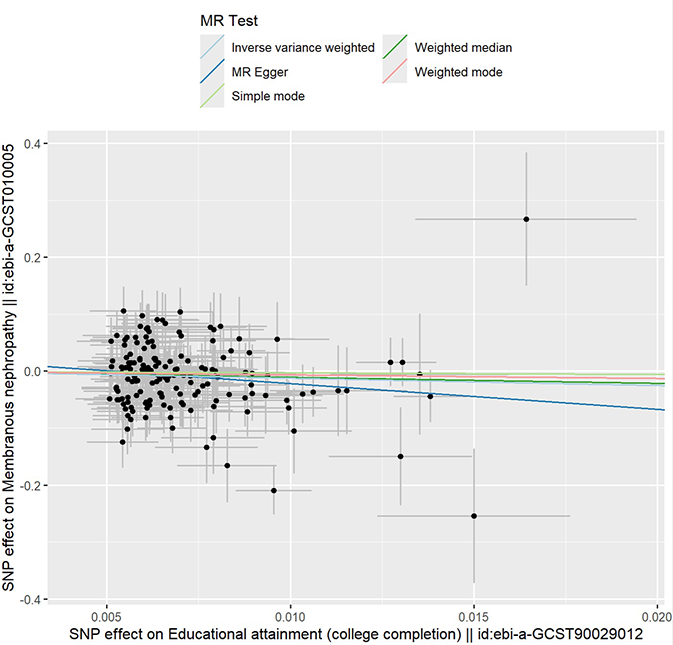

Supplement: Supplementary material picture 18.tif [file IRNF_A_2486620_SM8297.tif]

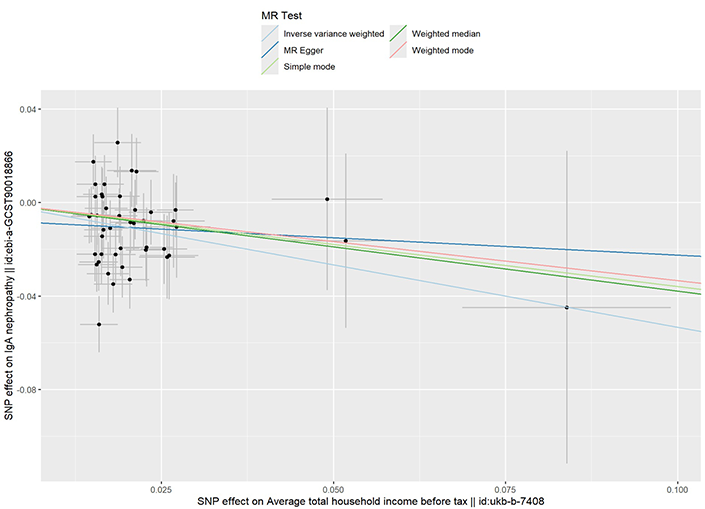

Supplement: Supplementary material picture 2.tif [file IRNF_A_2486620_SM8296.tif]

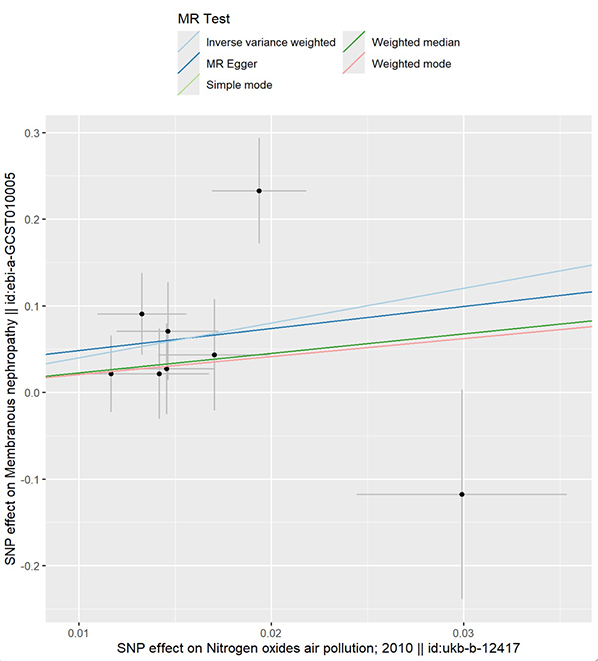

Supplement: Supplementary material picture 22.tif [file IRNF_A_2486620_SM8295.tif]

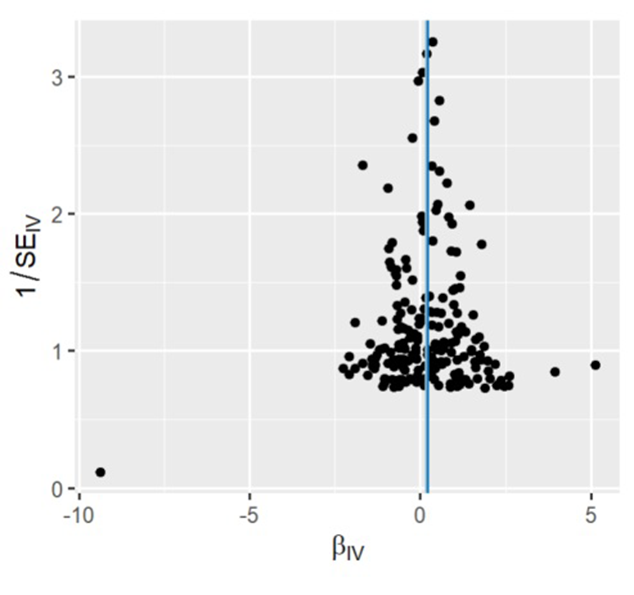

Supplement: Supplementary material picture 36.tif [file IRNF_A_2486620_SM8294.tif]

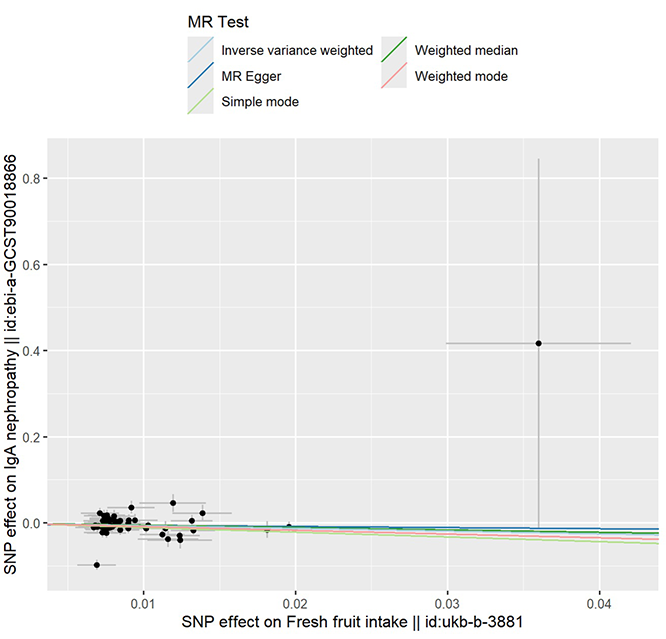

Supplement: Supplementary material picture 7.tif [file IRNF_A_2486620_SM8293.tif]

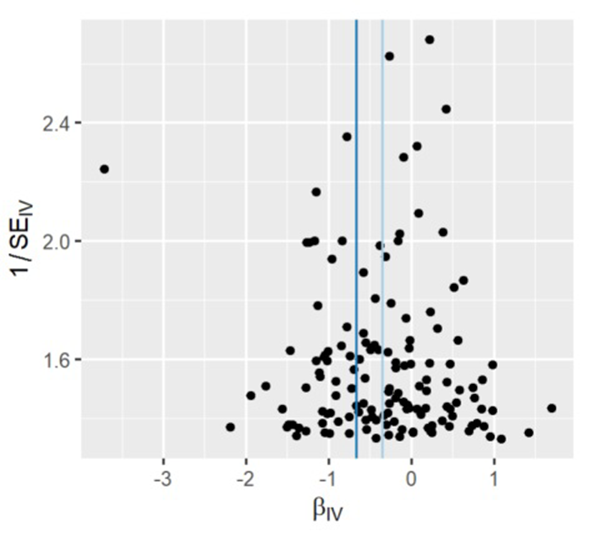

Supplement: Supplementary material picture 38.tif [file IRNF_A_2486620_SM8292.tif]

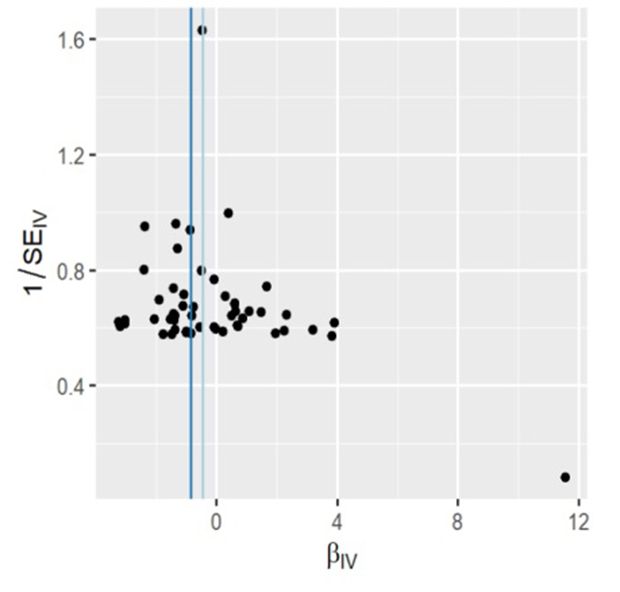

Supplement: Supplementary material picture 29.tif [file IRNF_A_2486620_SM8291.tif]

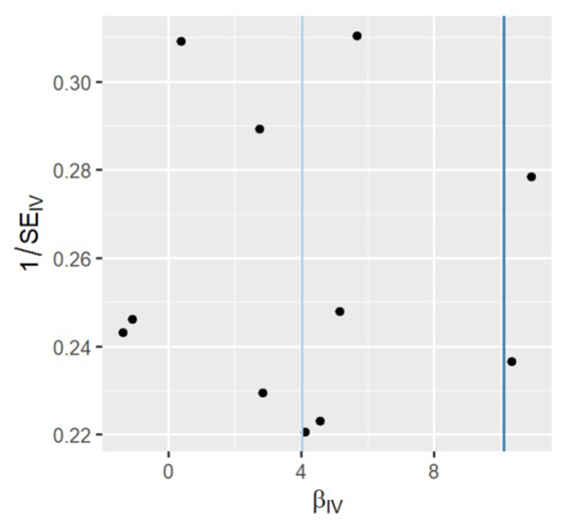

Supplement: Supplementary material picture 42.tif [file IRNF_A_2486620_SM8290.tif]

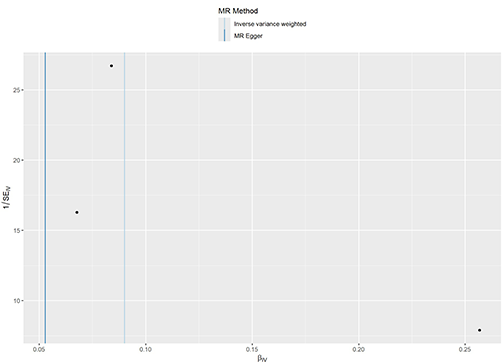

Supplement: Supplementary material picture 31.tif [file IRNF_A_2486620_SM8289.tif]
