# Supplementary material for: Causal associations between environmental factors and risk of IgA nephropathy and membranous nephropathy: a bidirectional Mendelian randomization and mediation analysis
Source: Ren Fail. 2025 Apr 9;47(1):2486620. doi: 10.1080/0886022X.2025.2486620 (PMC11983537; doi:10.1080/0886022X.2025.2486620)
Supplement: File 7.docx [file IRNF_A_2486620_SM8325.docx]

Supplementary material pictures 1 to 17 represent scatter plots of genetically predicted causal associations between educational attainment, average household economic income, frequency of alcohol consumption, insomnia, gluten-free diet, cheese, fresh fruit intake, triglycerides, transferrin saturation, waist circumference, hip circumference, percentage body fat, BMI, systolic blood pressure, diastolic blood pressure, cognitive performance, intelligence level and IgAN, respectively.

Supplementary material pictures 18 to 22 show scatter plots of genetically predicted causal relationships between educational attainment, moderate to vigorous physical activity levels, beef intake, waist-to-hip ratio adjusted for BMI, and nitrogen oxides air pollution and MN, respectively.

Supplementary material pictures 23 to 39 show funnel plots of genetically predicted causal relationships between educational attainment, mean household economic income, frequency of alcohol consumption, insomnia, gluten-free diet, cheese, fresh fruit intake, triglycerides, transferrin saturation, waist circumference, hip circumference, percentage body fat, body mass index, systolic blood pressure, diastolic blood pressure, cognitive ability, intelligence level and IgAN, respectively.

Supplementary material pictures 40 to 44 show funnel plots of the genetically predicted causal relationships between educational attainment, moderate to vigorous physical activity levels, beef intake, BMI-adjusted waist-to-hip ratio, and nitrogen oxides air pollution and MN, respectively.
