# Supplementary material for: Causal associations between environmental factors and risk of IgA nephropathy and membranous nephropathy: a bidirectional Mendelian randomization and mediation analysis
Source: Ren Fail. 2025 Apr 9;47(1):2486620. doi: 10.1080/0886022X.2025.2486620 (PMC11983537; doi:10.1080/0886022X.2025.2486620)
Supplement: File 1.docx [file IRNF_A_2486620_SM8322.docx]

**Supplementary table 1.** **STROBE-MR checklist.**

| **Item No.** | **Section** | **Checklist item** | **Page No.** | **Relevant text from manuscript** |
| --- | --- | --- | --- | --- |
| 1 | **TITLE and ABSTRACT** | Indicate Mendelian randomization (MR) as the study’s design in the title and/or the abstract if that is a main purpose of the study | 1 | Causal Associations Between Environmental Factors and Risk of IgA Nephropathy and Membranous Nephropathy: A Bidirectional Mendelian Randomization and Mediation Analysis |
|  | **INTRODUCTION** |  |  |  |
| 2 | **Background** | Explain the scientific background and rationale for the reported study. What is the exposure? Is a potential causal relationship between exposure and outcome plausible? Justify why MR is a helpful method to address the study question | 4 | Both external (e.g., pollutants, diet, lifestyle) and internal (e.g., metabolic factors, gut microbiome, hormones, inflammation) environmental exposures have been implicated in the incidence and progression of IgAN. The interaction between genetic susceptibility and environmental factors plays an important role in the pathogenesis of MN. Mendelian randomization (MR), utilizing genetic variation as an instrumental variable, is an effective new strategy for establishing causal relationships between exposure and outcome. |
| 3 | **Objectives** | State specific objectives clearly, including pre-specified causal hypotheses (if any). State that MR is a method that, under specific assumptions, intends to estimate causal effects | 2,4 | The purpose of this study was to use MR method to evaluate the causal association between five environmental exposures and two glomerular diseases, IgAN and MN. |
|  | **METHODS** |  |  |  |
| 4 | **Study design and data sources** | Present key elements of the study design early in the article. Consider including a table listing sources of data for all phases of the study. For each data source contributing to the analysis, describe the following: |  | See Figure 1 |
|  | a) | Setting: Describe the study design and the underlying population, if possible. Describe the setting, locations, and relevant dates, including periods of recruitment, exposure, follow-up, and data collection, when available. | 4-5 | In this two-sample MR study, we performed bidirectional, and two-step MR analyses to investigate the association between 5 types of environmental factors and IgAN and MN. The GWAS data for this study came from European populations. The original literature describes data sample collection, confounding control, and statistical methods, and ethical permission has been acquired. |
|  | b) | Participants: Give the eligibility criteria, and the sources and methods of selection of participants. Report the sample size, and whether any power or sample size calculations were carried out prior to the main analysis | 5 | Instrumental variables (IVs) for environmental factors and two glomerular diseases were obtained from the Integrative Epidemiology Unit ( IEU ) OpenGWAS database (IEU OpenGWAS project (mrcieu.ac.uk)) and the GWAS Catalog database (GWAS Catalog (ebi.ac.uk)). Statistics of primary MN traits were obtained from a GWAS, which included five European cohorts of 7979 individuals ( 2150 primary MN cases and 5829 controls ). The dataset of IgAN (GCST90018866) was comprised of 477,784 samples (15,587 IgAN cases and 462,197 controls). Detailed information is provided in supplementary material 2 |
|  | c) | Describe measurement, quality control and selection of genetic variants | 6 | Instrumental variable screening criteria: SNP with p-value < 5 × 10^-8^ and minor allele frequency (MAF) > 0.01 were extracted. The linkage disequilibrium (LD) SNPs were removed. The linkage disequilibrium (r²) was 0.001 and the genetic distance was 10,000 kb. Excluding weak IVs with F<10, F = R^2^ (n - k - 1) /k (1 - R2). Remove palindromic SNPs with unspecified allele frequencies or SNPs with incompatible alleles. |
|  | d) | For each exposure, outcome, and other relevant variables, describe methods of assessment and diagnostic criteria for diseases | 5 | MN: All primary MN cases were diagnosed by kidney biopsy, and any suspected cases secondary to autoimmune disease, drugs, infection, or malignancy were excluded.  IgAN: Diagnosis was confirmed in all IgAN patients by direct review of renal biopsy histopathology reports and clinical IgAN records.  Adjusted variables: These traits are detailed in previous studies. |
|  | e) | Provide details of ethics committee approval and participant informed consent, if relevant |  | Ethics approval and consent to participate is not applicable in this study. |
| 5 | **Assumptions** | Explicitly state the three core IV assumptions for the main analysis (relevance, independence and exclusion restriction) as well assumptions for any additional or sensitivity analysis | 6 | MR analyses were based on three basic assumptions: the instrumental variable SNPs were strongly linked with exposure factors, but not with other confounders, and they only influenced outcomes via the exposure pathway. |
| 6 | **Statistical methods: main analysis** | Describe statistical methods and statistics used |  |  |
|  | a) | Describe how quantitative variables were handled in the analyses (i.e., scale, units, model) |  | Figure2-4, Table1-3. OR. |
|  | b) | Describe how genetic variants were handled in the analyses and, if applicable, how their weights were selected | 6 | 1. SNPs must reach genome-wide significance P < 5×10^−8^ 2. Clumping technique 3. Harmonise |
|  | c) | Describe the MR estimator (e.g. two-stage least squares, Wald ratio) and related statistics. Detail the included covariates and, in case of two-sample MR, whether the same covariate set was used for adjustment in the two samples | 6-7 | The overall estimates using the IVW were used as main effects, with WM and MR- egger as complements. The bi-directional MR analysis was used to determine if there was reverse causality in the observed causal estimates. Two-step Mendelian randomization was used to assess mediation effects. |
|  | d) | Explain how missing data were addressed |  | No |
|  | e) | If applicable, indicate how multiple testing was addressed |  | No |
| 7 | **Assessment of assumptions** | Describe any methods or prior knowledge used to assess the assumptions or justify their validity | 6-7 | Additional sensitivity studies to evaluate the causal link between each group of exposures and outcome. Outliers were detected using leave-one-out analysis, and heterogeneity was assessed using the Cochran’s Q test. Where heterogeneity existed, it was evaluated using the IVW random effects model. The MR-Egger intercept method was used to test for horizontal pleiotropy. When p-value < 0.05, it indicates horizontal pleiotropy. The MR-Pleiotropy Residual Sum and Outlier (MR-PRESSO) was used to remove outlier SNPs and assesse whether excluding outlier SNPs affects causal estimations. |
| 8 | **Sensitivity analyses and additional analyses** | Describe any sensitivity analyses or additional analyses performed (e.g. comparison of effect estimates from different approaches, independent replication, bias analytic techniques, validation of instruments, simulations) | 6-7 | Outliers were detected using leave-one-out analysis, and heterogeneity was assessed using the Cochran’s Q test. Where heterogeneity existed, it was evaluated using the IVW random effects model. The MR-Egger intercept method was used to test for horizontal pleiotropy. When p-value < 0.05, it indicates horizontal pleiotropy. The MR-Pleiotropy Residual Sum and Outlier (MR-PRESSO) was used to remove outlier SNPs and assesse whether excluding outlier SNPs affects causal estimations. |
| 9 | **Software and pre-registration** |  |  |  |
|  | a) | Name statistical software and package(s), including version and settings used | 7 | All MR analyses were performed using the R package “TwoSampleMR”, “MRPRESSO”, “MVMR” in the R version 4.3.2. |
|  | b) | State whether the study protocol and details were pre-registered (as well as when and where) |  | No |
|  | **RESULTS** |  |  |  |
| 10 | **Descriptive data** |  |  |  |
|  | a) | Report the numbers of individuals at each stage of included studies and reasons for exclusion. Consider use of a flow diagram |  | supplementary material File 1 presents the characteristics of populations included in GWAS data on exposure and outcome. |
|  | b) | Report summary statistics for phenotypic exposure(s), outcome(s), and other relevant variables (e.g. means, SDs, proportions) | 5 | These traits are detailed in previous studies. |
|  | c) | If the data sources include meta-analyses of previous studies, provide the assessments of heterogeneity across these studies |  | Cochran's Q test indicated the heterogeneity. |
|  | d) | For two-sample MR:  i.  Provide justification of the similarity of the genetic variant-exposure associations between the exposure and outcome samples  ii.  Provide information on the number of individuals who overlap between the exposure and outcome studies | 5 | We used data from different sources to minimize sample overlap to ensure robustness. |
| 11 | **Main results** |  |  |  |
|  | a) | Report the associations between genetic variant and exposure, and between genetic variant and outcome, preferably on an interpretable scale |  | See supplementary material File 2 |
|  | b) | Report MR estimates of the relationship between exposure and outcome, and the measures of uncertainty from the MR analysis, on an interpretable scale, such as odds ratio or relative risk per SD difference |  | See Figures 2 to 4 |
|  | c) | If relevant, consider translating estimates of relative risk into absolute risk for a meaningful time period |  | No |
|  | d) | Consider plots to visualize results (e.g. forest plot, scatterplot of associations between genetic variants and outcome versus between genetic variants and exposure) |  | See supplementary material File 7 |
| 12 | **Assessment of assumptions** |  |  |  |
|  | a) | Report the assessment of the validity of the assumptions | 5 | Additionally, we conducted several sensitivity analyses to determine potential heterogeneity and horizontal pleiotropy. |
|  | b) | Report any additional statistics (e.g., assessments of heterogeneity across genetic variants, such as *I^2^*, Q statistic or E-value) |  | See Figures 2 to 4 |
| 13 | **Sensitivity analyses and additional analyses** |  |  |  |
|  | a) | Report any sensitivity analyses to assess the robustness of the main results to violations of the assumptions |  | See supplementary material File 5-7 |
|  | b) | Report results from other sensitivity analyses or additional analyses |  | See Figures 2 to 4 |
|  | c) | Report any assessment of direction of causal relationship (e.g., bidirectional MR) |  | See Figure 4 |
|  | d) | When relevant, report and compare with estimates from non-MR analyses |  | No |
|  | e) | Consider additional plots to visualize results (e.g., leave-one-out analyses) |  | See supplementary material File 7 and Figures 2 to 4 |
|  | **DISCUSSION** |  |  |  |
| 14 | **Key results** | Summarize key results with reference to study objectives | 5 | In this MR study, we comprehensively analyzed for the first time the causal relationship between 68 environmental factors and IgAN and MN. We identified 20 sets of causal associations, including 8 novel pairs and validated 12 previously reported pairs. This research provides a potential foundation for the establishment of precise etiological and risk assessment evidence for IgAN and MN. |
| 15 | **Limitations** | Discuss limitations of the study, taking into account the validity of the IV assumptions, other sources of potential bias, and imprecision. Discuss both direction and magnitude of any potential bias and any efforts to address them | 6 | Our analysis is subject to several inevitable limitations. First, potentially disease-related exposure factors reported in observational studies, such as silica and mercury, could not be assessed due to restrictions in GWAS data availability. Second, while limiting the study to a European population helped minimize demographic bias, it also constrained the generalizability of the MRI findings to other populations. Third, the existing GWAS data do not allow for stratified analysis to better investigate the impact of environmental factors in specific populations. |
| 16 | **Interpretation** |  |  |  |
|  | a) | Meaning: Give a cautious overall interpretation of results in the context of their limitations and in comparison with other studies | 11-15 | Discussion |
|  | b) | Mechanism: Discuss underlying biological mechanisms that could drive a potential causal relationship between the investigated exposure and the outcome, and whether the gene-environment equivalence assumption is reasonable. Use causal language carefully, clarifying that IV estimates may provide causal effects only under certain assumptions | 11-15 | Discussion |
|  | c) | Clinical relevance: Discuss whether the results have clinical or public policy relevance, and to what extent they inform effect sizes of possible interventions | 11-15 | Discussion |
| 17 | **Generalizability** | Discuss the generalizability of the study results (a) to other populations, (b) across other exposure periods/timings, and (c) across other levels of exposure |  | NO |
|  | **OTHER INFORMATION** |  |  |  |
| 18 | **Funding** | Describe sources of funding and the role of funders in the present study and, if applicable, sources of funding for the databases and original study or studies on which the present study is based | 16 | This study was supported by the Project of Health Commission of Hubei Province (grant number WJ2019Q001) and the Project of Wuhan Science and Technology (grant number 2019020701011434). |
| 19 | **Data and data sharing** | Provide the data used to perform all analyses or report where and how the data can be accessed, and reference these sources in the article. Provide the statistical code needed to reproduce the results in the article, or report whether the code is publicly accessible and if so, where | 5 | the Integrative Epidemiology Unit ( IEU ) OpenGWAS database (IEU OpenGWAS project (mrcieu.ac.uk)) and the GWAS Catalog database (GWAS Catalog (ebi.ac.uk)) |
| 20 | **Conflicts of Interest** | All authors should declare all potential conflicts of interest | 16 | The authors declare that they have no known competing financial interests or personal relationships that could have appeared to influence the work reported in this paper. |

**Supplementary Table 2. Summary of the GWAS datasets involved in the Mendelian randomization investigation**

| Classification | GWAS ID | Sample size | Main consortium/cohort | Unit | Author (year) | PubMed ID |
| --- | --- | --- | --- | --- | --- | --- |
| Educational attainment | ebi-a-GCST90029012 | 470,941 | UK Biobank | - | Loh PR (2018) | 29892013 |
| Household income | ukb-b-7408 | 397,751 | MRC-IEU | SD | Elsworth(2018) | - |
| Loneliness | ukb-b-8476 | 455,364 | MRC-IEU | SD | Elsworth(2018) | - |
| Age of initiation of regular smoking | ieu-b-24 | 341,427 | GWAS and Sequencing Consortium of Alcohol and Nicotine use | - | Liu, M(2019) | 30643251 |
| Cigarettes per Day | ieu-b-25 | 337,334 | GWAS and Sequencing Consortium of Alcohol and Nicotine use | - | Liu, M(2019) | 30643251 |
| Tobacco smoking: Never smoked | ukb-d-22506_114 | 91,353 | - | - | Neale lab(2018) | - |
| Alcoholic drinks per week | ieu-b-73 | 335,394 | GWAS and Sequencing Consortium of Alcohol and Nicotine use | - | Liu, M(2019) | 30643251 |
| Alcohol intake frequency | ukb-b-5779 | 462,346 | MRC-IEU | SD | Elsworth(2018) | - |
| Average weekly spirits intake | ukb-b-1707 | 326,565 | MRC-IEU | SD | Elsworth(2018) | - |
| Average weekly beer plus cider intake | ukb-b-5174 | 327,634 | MRC-IEU | SD | Elsworth(2018) | - |
| Average weekly red wine intake | ukb-b-5239 | 327,634 | MRC-IEU | SD | Elsworth(2018) | - |
| Processed meat intake | ukb-b-6324 | 461,981 | MRC-IEU | SD | Elsworth(2018) | - |
| Beef intake | ukb-b-2862 | 461,053 | MRC-IEU | SD | Elsworth(2018) | - |
| Poultry intake | ukb-b-8006 | 461,900 | MRC-IEU | SD | Elsworth(2018) | - |
| Oily fish intake | ukb-b-2209 | 460,443 | MRC-IEU | SD | Elsworth(2018) | - |
| non-oily fish intake | ukb-b-17627 | 460,880 | MRC-IEU | SD | Elsworth(2018) | - |
| Lamb/mutton intake | ukb-b-14179 | 460,006 | MRC-IEU | SD | Elsworth(2018) | - |
| cheese intake | ukb-b-1489 | 451,486 | MRC-IEU | SD | Elsworth(2018) | - |
| Bread intake | ukb-b-11348 | 452,236 | MRC-IEU | SD | Elsworth(2018) | - |
| Tea intake | ukb-b-6066 | 447,485 | MRC-IEU | SD | Elsworth(2018) | - |
| Salad / raw vegetable intake | ukb-b-1996 | 435,435 | MRC-IEU | SD | Elsworth(2018) | - |
| Cooked vegetable intake | ukb-b-8089 | 448,651 | MRC-IEU | SD | Elsworth(2018) | - |
| Cereal intake | ukb-b-15926 | 441,640 | MRC-IEU | SD | Elsworth(2018) | - |
| Gluten-free | ukb-b-11189 | 64,949 | MRC-IEU | SD | Elsworth(2018) | - |
| Fresh fruit intake | ukb-b-3881 | 446,462 | MRC-IEU | SD | Elsworth(2018) | - |
| Dried fruit intake | ukb-b-16576 | 421,764 | MRC-IEU | SD | Elsworth(2018) | - |
| coffee intake | ukb-b-5237 | 428,860 | MRC-IEU | SD | Elsworth(2018) | - |
| Sleep duration | ukb-b-4424 | 460,099 | MRC-IEU | SD | Elsworth(2018) | - |
| Morning person | ebi-a-GCST90029029 | 452,896 | - | - | Loh PR(2018) | 29892013 |
| Sleeplessness / insomnia | ukb-b-3957 | 462,341 | MRC-IEU | SD | Elsworth(2018) | - |
| Moderate to vigorous physical activity levels | ebi-a-GCST006097 | 377,234 | - | - | Klimentidis(2018) | 29899525 |
| Vigorous physical activity | ebi-a-GCST006098 | 261,055 | - | - | Klimentidis(2018) | 29899525 |
| Strenuous sports or other exercises | ebi-a-GCST006100 | 350,492 | - | - | Klimentidis(2018) | 29899525 |
| HbA1c | ebi-a-GCST90014006 | 389,889 | - | - | Mbatchou J(2021) | - |
| Two-hour glucose | ebi-a-GCST90002227 | 63,396 | - | - | Chen J(2021) | 34059833 |
| Fasting glucose | ebi-a-GCST90002232 | 200,622 | - | - | Chen J(2021) | 34059833 |
| Fasting insulin | ebi-a-GCST90002238 | 151,013 | - | - | Chen J(2021) | 34059833 |
| LDL cholesterol | ieu-b-5089 | 201,678 | UK Biobank | SD | Si Fang(2022) | - |
| HDL cholesterol levels | ebi-a-GCST90025956 | 400,754 | - | - | Barton AR | 34226706 |
| Triglycerides | ebi-a-GCST90018975 | 343,992 | - | - | Sakaue S (2021) | 34594039 |
| Apolipoprotein A1 levels | ebi-a-GCST90025955 | 398,508 | - | - | Barton AR(2021) | 34226706 |
| Apolipoprotein B levels | ebi-a-GCST90025952 | 398,508 | - | - | Barton AR(2021) | 34226706 |
| Transferrin | ieu-a-1052 | 23,986 | GIS | SD | Benyamin(2014) | 25352340 |
| Serotransferrin measurement | ebi-a-GCST90019443 | 10,708 | - | - | Pietzner M(2020) | 33328453 |
| Transferrin Saturation | ieu-a-1051 | 23,986 | GIS | SD | Benyamin(2014) | 25352340 |
| ferritin | ieu-b-5115 | 246,139 | - | SD | Bell(2021) | 10.1038/s42003-020-01575-z(DOI) |
| Ferritin | prot-a-1148 | 3,301 | - | - | Sun BB(2018) | 29875488 |
| Iron | ieu-a-1049 | 23,986 | GIS | - | Benyamin(2014) | 25352340 |
| Serum uric acid levels | ebi-a-GCST90018977 | 343,836 | - | - | Sakaue S(2021) | 34594039 |
| Calcium levels | ebi-a-GCST90025990 | 400,792 | - | - | Barton AR(2021) | 34226706 |
| Alkaline phosphatase levels | ebi-a-GCST90013991 | 389,883 | - | - | Mbatchou J(2021) | 34017140 |
| Serum 25-Hydroxyvitamin D levels | ebi-a-GCST90000618 | 496,946 | - | - | Revez JA(2020) | 32242144 |
| FEV1 | ebi-a-GCST007432 | 321,047 | - | - | Shrine N(2019) | 30804560 |
| Peak expiratory flow | ebi-a-GCST007430 | 321,047 | - | - | Shrine N(2019) | 30804560 |
| Forced vital capacity (FVC) | ukb-b-7953 | 421,986 | MRC-IEU | SD | Ben Elsworth(2018) | - |
| Lung function (FEV1/FVC) | ebi-a-GCST90029026 | 421,986 | MRC-IEU | SD | Ben Elsworth(2018) | - |
| Cognitive performance | ebi-a-GCST006572 | 257,841 | - | - | Lee JJ(2018) | 30038396 |
| Intelligence | ebi-a-GCST006250 | 269,867 | - | - | Savage JE(2018) | 29942086 |
| Waist circumference | ukb-b-9405 | 462,166 | MRC-IEU | SD | Ben Elsworth(2018) | - |
| Body fat percentage | ukb-b-8909 | 454,633 | MRC-IEU | SD | Ben Elsworth(2018) | - |
| Body mass index (BMI) | ukb-b-19953 | 461,460 | MRC-IEU | SD | Ben Elsworth(2018) | - |
| Hip circumference | ukb-b-15590 | 462,117 | MRC-IEU | SD | Ben Elsworth(2018) | - |
| Waist-to-hip ratio adjusted for BMI | ebi-a-GCST90025996 | 458,349 | - | - | Barton AR(2021) | 34226706 |
| Visceral adipose tissue volume | ebi-a-GCST90016671 | 32,860 | - | - | Liu Y(2021) | 34128465 |
| Systolic blood pressure | ebi-a-GCST90000062 | 810,865 | - | - | Surendran P(2020) | 33230300 |
| Diastolic blood pressure | ebi-a-GCST90000063 | 810,865 | - | - | Surendran P(2020) | 33230300 |
| Particulate matter air pollution (pm2.5) | ukb-b-10817 | 423,796 | MRC-IEU | SD | Ben Elsworth(2018) | - |
| Particulate matter air pollution (pm10); 2010 | ukb-b-18469 | 423,796 | MRC-IEU | SD | Ben Elsworth(2018) | - |
| Nitrogen oxides air pollution; 2010 | ukb-b-12417 | 456,380 | MRC-IEU | SD | Ben Elsworth(2018) | - |
| Nitrogen dioxide air pollution; 2010 | ukb-b-9942 | 456,380 | MRC-IEU | SD | Ben Elsworth(2018) | - |
| IgA nephropathy | ebi-a-GCST90018866 | 477,784 | UK Biobank and FinnGen | - | Sakaue S(2021) | 34594039 |
| Membranous nephropathy | ebi-a-GCST010005 | 7,979 | - | - | Xie J(2020) | 32231244 |

BMI, body mass index; GWAS, genome-wide association study; HbA1c, glycated hemoglobin; HDL, high-density lipoprotein; LDL, low-density lipoprotein; GIS, the Genetics of Iron Status Consortium; MRC-IEU, MRC Integrative Epidemiology Unit; SD, standard deviation; UKB, UK Biobank; 2hGlu, 2h-glucose post-challenge; 25OHD, 25 hydroxyvitamin D. FEV1, forced expiratory volume in one second.
