# Supplementary material for: Causal associations between environmental factors and risk of IgA nephropathy and membranous nephropathy: a bidirectional Mendelian randomization and mediation analysis
Source: Ren Fail. 2025 Apr 9;47(1):2486620. doi: 10.1080/0886022X.2025.2486620 (PMC11983537; doi:10.1080/0886022X.2025.2486620)
Supplement: List of supplementary material.docx [file IRNF_A_2486620_SM8288.docx]

List of supplementary material

| File 1 | STROBE-MR checklist and Summary of the GWAS datasets involved |
| --- | --- |
| File 2 | Harmonised instrumental variables |
| File 3 | Statistically significant from Mendelian randomization analysis |
| File 4 | Instrumental variables used in Mendelian randomization analysis |
| File 5 | Results of the Leave-one-out method |
| File 6 | Effect values of single SNPs |
| File 7 | Statistically significant scatterplots and funnel plots |
